# Two transcriptionally and functionally distinct waves of neutrophils during mouse acute liver injury

**DOI:** 10.1097/HC9.0000000000000459

**Published:** 2024-06-19

**Authors:** Yousef Maali, Manuel Flores Molina, Omar Khedr, Mohamed N. Abdelnabi, Jessica Dion, Ghada S. Hassan, Naglaa H. Shoukry

**Affiliations:** 1 Immunopathology Axis, Centre de Recherche du Centre hospitalier de l’Université de Montréal (CRCHUM), Montréal, Quebec, Canada; 2 Département de microbiologie, infectiologie et immunologie, Université de Montréal, Montréal, Quebec, Canada; 3 Departement de médecine, Université de Montréal, Montréal, Quebec, Canada

## Abstract

**Background:**

Neutrophils are key mediators of inflammation during acute liver injury (ALI). Emerging evidence suggests that they also contribute to injury resolution and tissue repair. However, the different neutrophil subsets involved in these processes and their kinetics are undefined. Herein, we characterized neutrophil kinetics and heterogeneity during ALI.

**Methods:**

We used the carbon tetrachloride model of ALI and employed flow cytometry, tissue imaging, and quantitative RT-PCR to characterize intrahepatic neutrophils during the necroinflammatory early and late repair phases of the wound healing response to ALI. We FACS sorted intrahepatic neutrophils at key time points and examined their transcriptional profiles using RNA-sequencing. Finally, we evaluated neutrophil protein translation, mitochondrial function and metabolism, reactive oxygen species content, and neutrophil extracellular traps generation.

**Results:**

We detected 2 temporarily distinct waves of neutrophils during (1) necroinflammation (at 24 hours after injury) and (2) late repair (at 72 hours). Early neutrophils were proinflammatory, characterized by: (1) upregulation of inflammatory cytokines, (2) activation of the noncanonical NF-κB pathway, (3) reduction of protein translation, (4) decreased oxidative phosphorylation, and (5) higher propensity to generate reactive oxygen species and neutrophil extracellular traps. In contrast, late neutrophils were prorepair and enriched in genes and pathways associated with tissue repair and angiogenesis. Finally, early proinflammatory neutrophils were characterized by the expression of a short isoform of C-X-C chemokine receptor 5, while the late prorepair neutrophils were characterized by the expression of C-X-C chemokine receptor 4.

**Conclusions:**

This study underscores the phenotypic and functional heterogeneity of neutrophils and their dual role in inflammation and tissue repair during ALI.

## Graphical abstract

**Figure:**
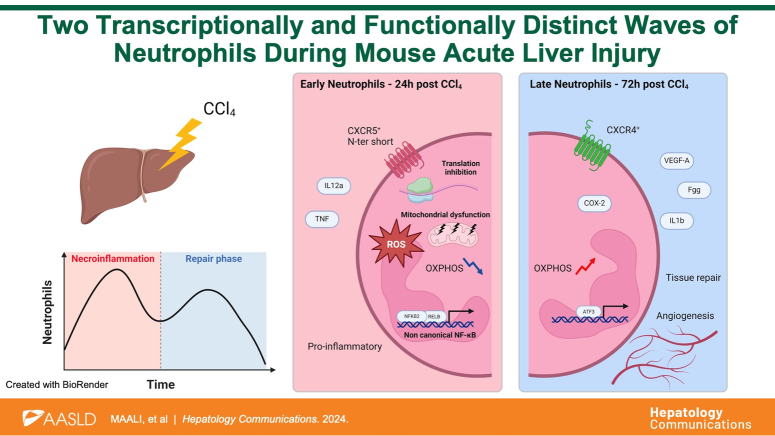

## INTRODUCTION

Acute liver injury (ALI) of various etiologies is characterized by severe hepatic necrosis, inflammation, and tissue damage, and may progress to acute liver failure associated with high morbidity and mortality.^1^ Neutrophils play a pivotal role in the initial phases of inflammation during ALI.^2^ Emerging evidence suggests that neutrophils could also coordinate inflammation resolution and contribute to tissue repair in many organs including the liver.^3^

Recent studies have highlighted the remarkable heterogeneity of neutrophils, prompting investigations into the distinct subpopulations that infiltrate the liver during different stages of liver injury.^4^ Advancements in single-cell transcriptomics have unveiled the presence of proinflammatory (N1) and anti-inflammatory (N2) neutrophil subsets, with distinct gene expression profiles and functional characteristics.^5,6^ During tissue repair, neutrophils enhance the breakup of damaged vessels, the assembly of new ones, and the remodeling of extracellular matrix upon focal thermal sterile injury of the liver, enabling damage resolution.^7^ Moreover, reactive oxygen species (ROS) released from neutrophils promote tissue repair by enhancing the differentiation of the proinflammatory monocytes/macrophages (Ly6C^hi^CX3CR1^−^) into proresolving macrophages (Ly6C^lo^CX3CR1^+^) as demonstrated in acetaminophen-induced ALI.^8^ Depleting neutrophils were associated with both improvement and worsening liver injuries, implying fine regulation of different neutrophil subtypes with distinct functions.^9^ Using longitudinal flow cytometry analysis, we have previously observed 2 waves of neutrophils infiltrating the liver following carbon tetrachloride (CCl_4_)–induced ALI during the necroinflammatory and repair phases, respectively, suggesting that they may be functionally distinct.^10^ Yet, it remains unclear how different neutrophil subsets are recruited to the liver during various stages of liver injury and the molecular pathways that govern their different functions remain unexplored. To address these questions, we studied the kinetics, phenotype, transcriptomic profile, and functions of neutrophils during CCl_4_-induced ALI. We report 2 transcriptionally and functionally distinct neutrophil populations peaking either early during the inflammation phase or late during the repair phase.

## METHODS

### Animals

Eight- to 10-week-old C57BL/6N male mice (Strain code 027; Charles River Laboratories, Senneville, QC, Canada) were maintained in a specific pathogen-free facility at the Centre de Recherche du Centre hospitalier de l’Université de Montréal (CRCHUM). All procedures were approved by the Institutional Animal Care and Use Committee (Protocol IP18035NSs). Mice received a single i.p. injection of CCl_4_ (Sigma-Aldrich, Oakville, ON, Canada) at 1 mL/kg in corn oil (Sigma-Aldrich). Mice were terminally euthanized at 0, 12, 24, 48, 72, 96, and 168 hours after injection. Spleens from untreated mice were used to isolate control neutrophils for RNA-sequencing (RNA-seq).

### Isolation of neutrophils

Intrahepatic leukocytes were isolated through a two-step process, involving mechanical dissociation of the liver followed by enzymatic digestion and purification on a Percol gradient (37%) (MilliporeSigma, Burlington, MA). Splenocytes were isolated by mechanical dissociation of the spleen and passed through a 70-µm filter. Liver and spleen neutrophils were enriched using an anti-Ly6G microbead mouse kit (Miltenyi-Biotec, Bergisch Gladbach, Germany). Details are provided in the Supplemental Section, http://links.lww.com/HC9/A915.

### RNA-Seq library preparation

Enriched neutrophils were FACS sorted as Ly6G^hi^Ly6C^int^CD11b^+^CD45^+^ (>95% purity) directly in lysis buffer. RNA was extracted using an RNeasy micro-kit (Qiagen, Germantown, MD). RNA quality was assessed using the Agilent Bioanalyzer 2100 system (Agilent Technologies, Santa Clara, CA). Only RNA samples displaying an RNA integrity number score >7 were used. Library preparation and sequencing were performed by the Genome Quebec Innovation Center (Montreal, QC, Canada). Libraries were prepared using the New England Biolabs mRNA stranded library Prep Kit (New England Biolabs, Ipswich, MA), then sequenced on the Illumina NovaSeq PE100—25M (Illumina Inc., San Diego, CA). Reads obtained were already aligned to the mice genome (GRCm38.x). Read counts per gene were determined using HTSeq-count (v0.6.1p1). Details about bioinformatic analysis are provided in the Supplemental Section, http://links.lww.com/HC9/A915.

### Flow cytometry

Leukocytes were washed with FACS buffer (PBS, 1% fetal bovine serum, 0.02% sodium azide), then stained with surface antibodies plus aqua vivid and 10% mouse serum in 96-well plates for 30 minutes at 4 °C in the dark, and then washed twice in FACS buffer. Cell populations were defined as: neutrophils, CD11b^+^Ly6C^int^Ly6G^+^; monocytes, CD11b^+^Ly6C^hi^Ly6G^−^; macrophages, CD11b^+^Ly6C^lo/−^Ly6G^-^Siglec-F^−^F4/80^+^; dendritic cells, CD11b^+^Ly6C^−^Ly6G^−^CD11c^+^; eosinophils, CD11b^+^F4/80^int/+^Siglec-F^+^; and T cells, CD11b^−^CD3^+^. Samples were acquired on a BD LSRFortessa Cytometer equipped with violet (405 nm), blue (488 nm), yellow-green (561 nm), and red (633 nm) lasers, utilizing FACSDiva version 8.0.1 (BD Biosciences, San Diego, CA). Data were analyzed using FlowJo version 10.4 for Mac (BD Biosciences). Antibodies used are listed in Supplemental Table S1, http://links.lww.com/HC9/A915.

### Immunofluorescence and quantification

Immunofluorescence (IF) for myeloperoxidase was performed on 4-mm formalin-fixed, paraffin-embedded tissue sections. Image analysis and quantification were performed utilizing the Visiopharm image analysis software (Visiopharm, Hoersholm, Denmark) as described.^11^ Multiplex immunofluorescence for ionized calcium binding adaptor molecule 1, alpha smooth muscle actin, Desmin, and myeloperoxidase was done on frozen 5-μm optimal cutting temperature compound-embedded and fixed liver sections, as described.^10^ Antibodies used for IF are listed in Supplemental Table S2, http://links.lww.com/HC9/A915.

### RNA isolation and reverse transcription polymerase chain reaction analysis

Total liver RNA was isolated using RNeasy Mini Kit (Qiagen). Complementary DNA (cDNA) was generated from 2 µg of total RNA using the Transcriptor Universal cDNA Master Mix (Roche Life Science, Penzberg, Germany). For neutrophils, cDNA was generated from 200 ng of total RNA isolated from FACS-sorted neutrophils using the RNeasy micro-kit (Qiagen). cDNA was amplified using the LightCycler 480 SYBR Green I Master (Roche) in the LightCycler 480 instrument (Roche) according to the manufacturer’s protocols. mRNA expression was normalized to the expression of the housekeeping gene 28s and was determined using the 2-ΔΔCt method. Primer sequences are listed in Supplemental Table S3, http://links.lww.com/HC9/A915.

### Mitochondrial functionality assay

Freshly isolated neutrophils were seeded at 1.5×10^5^ cells per well in 96-well round bottom plates. Cells were incubated with surface antibodies and a mixture of 50-nM MitoTracker Green FM and 50-nM MitoTracker Red CMXRos (both from Thermo Fisher Scientific, Waltham, MA) for 15 minutes at 37 °C. Cells were then washed and directly analyzed by flow cytometry. Details are provided in the Supplemental Section, http://links.lww.com/HC9/A915.

### Puromycin incorporation assay

Cells (1.5×10^5^/well) seeded in 96-well round bottom plates were incubated in 150 µL Roswell Park Memorial Institute complete medium+ 10% fetal bovine serum containing puromycin (10 µg/mL) with or without cycloheximide (100 µg/mL) for 30 minutes at 37 °C. Cells were then washed and stained for surface and viability markers for 30 minutes at 4 °C, then permeabilized using BD Cytofix/Cytoperm (BD) for 30 minutes at 4 °C in the dark, after which cells were washed and incubated for 30 minutes at 4 °C in the dark with the A-647-labeled anti-puromycin antibody, then analyzed by flow cytometry. Details are provided in the Supplemental Section, http://links.lww.com/HC9/A915.

### Real-time bioenergetic profile analysis

Mito Stress Test was performed using the Seahorse XFe96 Analyser (Agilent Technologies) at the Metabolomics Innovation Resource instrumentation research platform, McGill University (Montreal, QC, Canada). FACS-sorted neutrophils suspended in complete Roswell Park Memorial Institute were seeded at 300,000 neutrophils per well in XFe96 cell culture microplates (Agilent Technologies) precoated with poly-L-lysine. Plates were centrifuged at 200*g* for 1 minute with no brakes. Roswell Park Memorial Institute medium was replaced with 180 μL of DMEM XF base medium, pH 7.4 (Agilent Technologies), supplemented with 10 mM glucose, 2 mM glutamine, and 1 mM pyruvate (Agilent Technologies). Plates were incubated for 30 minutes at 37 °C and loaded into the Seahorse XFe96 Analyzer. In the analyzer, 2 µM oligomycin, 1 µM carbonyl cyanide-4-(trifluoromethoxy) phenylhydrazone, and 0.5 µM rotenone + antimycin A were injected at the indicated times. Respiratory parameters were collected.

### Statistical analysis

Statistical analysis was performed with GraphPad Prism 9 software. Mann-Whitney test was used to determine the significance between 2 groups. ANOVA followed by Dunn Multiple Comparison Test was used when comparing more than 2 groups. A *p*-value <0.05 was considered statistically significant.

## RESULTS

### Two neutrophil peaks during necroinflammation and tissue repair after CCl_4_-induced ALI

**FIGURE 1:**
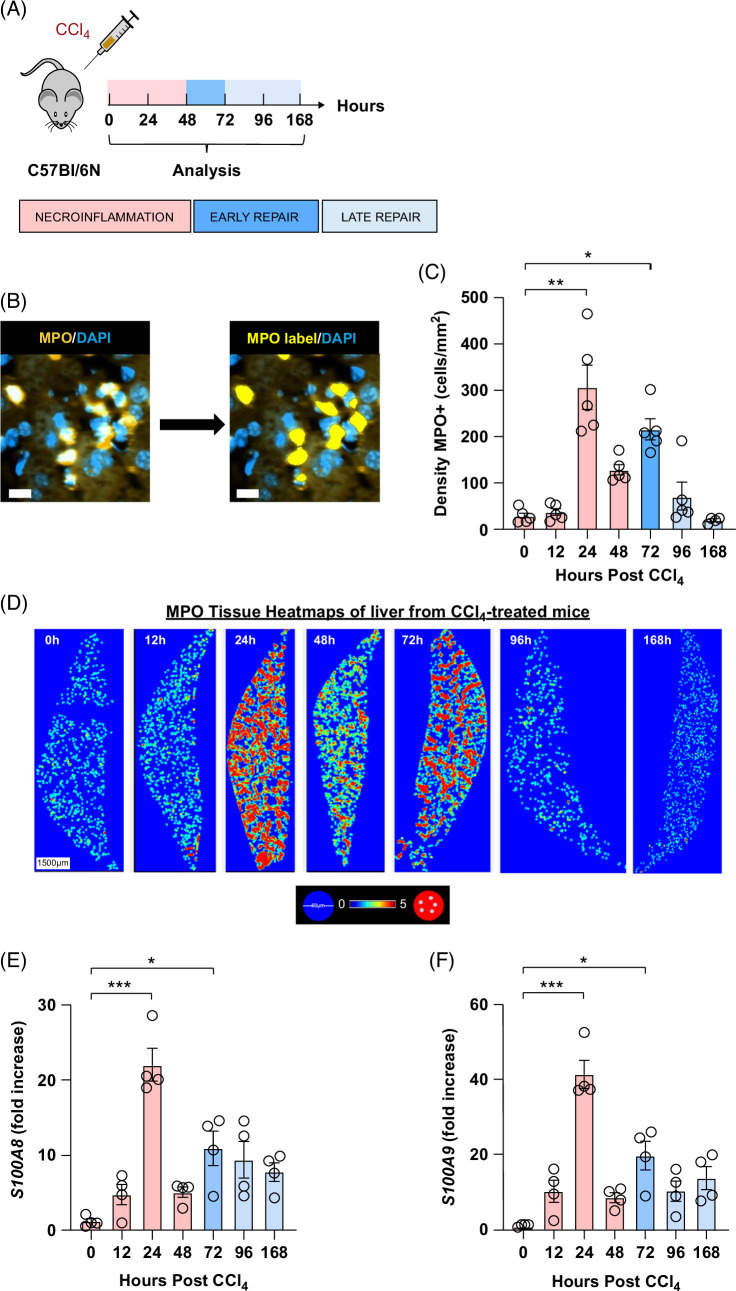
CCl_4_ toxicity induces two waves of neutrophils during necroinflammation and tissue repair. (A) Schematics of the experimental design delineating the phases of the wound healing response to 1 i.p. injection of CCl_4_ at 1 mL/kg of body weight. (B) Representative staining of intrahepatic neutrophils (MPO^+^). (C) Density of intrahepatic neutrophils expressed as the number of MPO^+^ cells per mm^2^. Image analysis was performed using the image analysis software VIS (Visiopharm). (D) Representative tissue heatmaps of MPO^+^ cells, scale bar = 1500 μm. High-density areas are displayed in red, low-density areas are displayed in blue, and intermediate values are displayed according to the color scale in the figure. Relative gene expression of (E) *S100A8* and (F) *S100A9* determined by quantitative PCR on bulk liver tissue. The mRNA expression data represent fold increase relative to 0-h controls and was normalized to 28 seconds. N = 4–5 mice per group. Data are shown as mean±SEM. Statistical analysis was performed using one-way ANOVA on ranks followed by Dunn Multiple Comparison Test. *p<0.05, **p<0.01, ***p<0.001. Abbreviations: CCl_4_, carbon tetrachloride; MPO, myeloperoxidase.

To investigate the kinetics of neutrophils during CCl_4_-induced ALI, C57BL/6N mice received a single i.p. injection of CCl_4_ (1 mL/kg of body weight), and liver tissue was analyzed at various time points afterward. As we previously described using histological and functional characterization, we delineated 3 different stages of liver injury: (1) necroinflammation (0–48 hours), (2) early repair (48–72 hours), and (3) late repair (72–168 hours)^10^ (Figure 1A and Supplemental Figure S1A, B, http://links.lww.com/HC9/A915).

**FIGURE 2:**
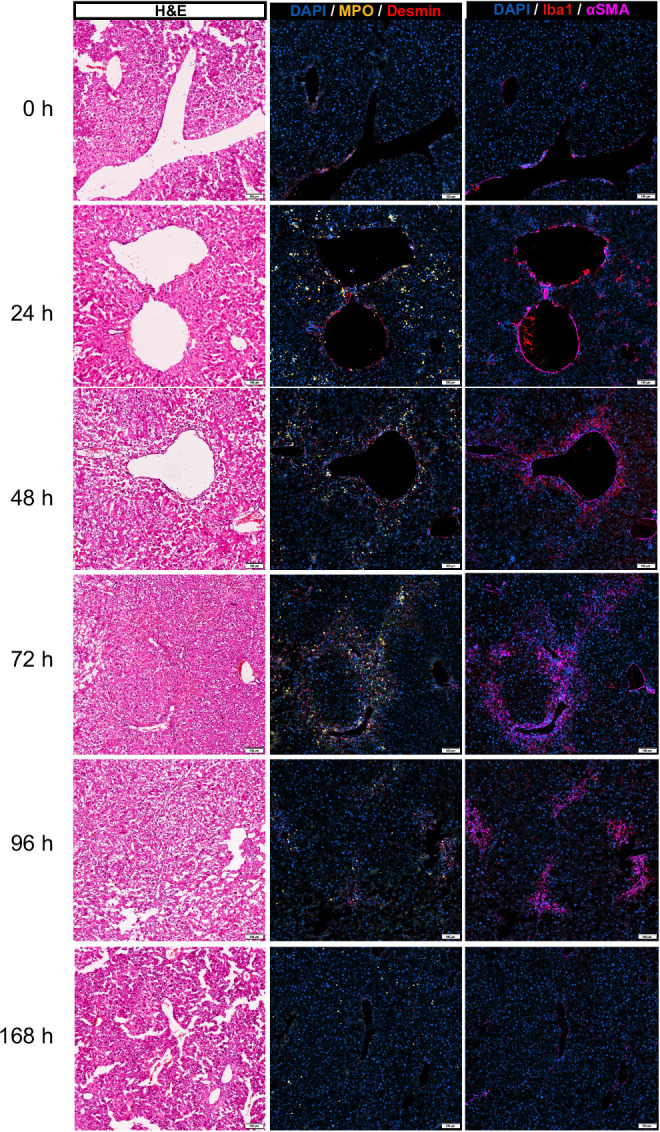
MPO^+^ cells are distributed throughout the liver tissue at 24 hours post-CCl_4_ but localize in the repair area at 72 hours. Representative immunofluorescence images demonstrating the location of MPO^+^ cells relative to activated macrophages (Iba1^+^) and activated HSCs (αSMA^+^/Desmin^+^) at the different phases of the wound healing response. Aligned serial sections of hepatic tissue: section 1 (H&E), section 2 (MPO and Desmin), and section 3 (Iba1 and αSMA). Scale bar = 100 μm. Abbreviations: CCl_4_, carbon tetrachloride; H&E, Hematoxylin and eosin; IBA1, ionized calcium binding adaptor molecule 1; MPO, myeloperoxidase; αSMA, alpha smooth muscle actin.

We investigated neutrophil infiltration into the liver by the quantification of MPO^+^ cells on whole liver sections using IF. The number of neutrophils in the liver was minimal at 0 hours. During the necroinflammation phase (24 hours post-CCl_4_), there was an extensive influx of neutrophils (Figure 1B–D), hereinafter termed 24-hour neutrophils. Neutrophil density dropped at 48 hours followed by a distinct second wave of neutrophils at early repair (72 hours post-CCl_4_), hereinafter termed 72-hour neutrophils. This confirmed our previous observation using flow cytometry^10^ (Supplemental Figure S1C, D, http://links.lww.com/HC9/A915). To further dissect the role of these different waves of neutrophils and evaluate their individual localization, we examined the interactions between neutrophils and various cellular populations using IF on liver sections. We observed a distinct distribution of neutrophils throughout the progression of ALI. At 24 hours after injury, neutrophils identified as MPO^+^ cells infiltrated both necrotic and non-necrotic regions within the liver tissue. However, by 48–72 hours after injury, these MPO^+^ cells predominantly localized to regions indicative of repair, characterized by the presence of activated macrophages (ionized calcium binding adaptor molecule 1^+^), and activated HSCs (alpha smooth muscle actin^+^/Desmin^+^). Such differential distribution suggests a prorepair role of 72-hour neutrophils (Figure 2).

Finally, we evaluated changes in gene expression of the inflammatory markers *S100a8* and *S100a9* that correlate with neutrophil abundance at sites of inflammation.^12^ Concomitant with the liver myeloperoxidase signals, we observed 2 high expression peaks of *S100a8* and *S100a9* at 24 hours and 72 hours post-CCl_4_ (Figure 1E, F). In summary, we detected 2 intrahepatic neutrophil waves with distinct distribution during the necroinflammation and tissue repair phases of ALI, respectively.

### The first and second waves of neutrophils exhibit distinct transcriptomic profiles

**FIGURE 3:**
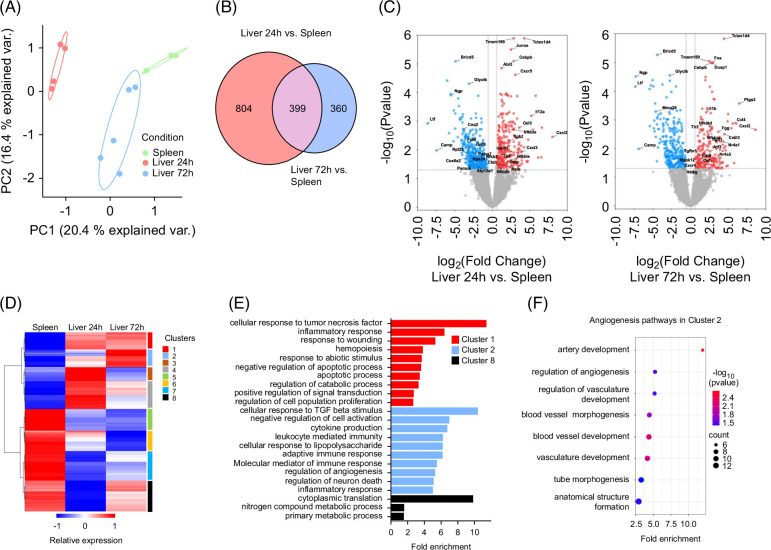
Neutrophils from 24 hours and 72 hours after injury exhibit distinct transcriptomic profiles. Bulk RNA-seq analysis was conducted on FACS-sorted CD45^+^CD11b^+^Ly6G^+^Ly6C^int^ neutrophils from the spleen (control) and liver at 24 hours and 72 hours post-carbon tetrachloride. (A) Principal component analysis performed on the normalized data of neutrophils isolated from the spleen (green) and liver 24 hours (red) and 72 hours post-CCl_4_ (blue). (B) Venn diagram of unique and overlapping differentially expressed genes (DEGs) between contrasts Liver 24-hour versus spleen and liver 72-hour versus spleen. (C) Volcano plots showing DEGs (bulk RNA-seq data) of liver 24-hour versus spleen (left) and liver 72-hour versus spleen (right), respectively. The *x*-axis represents the log2 Fold change and the *y*-axis represents the −log_10_ adjusted *p*-values (≤0.05). Dotted lines indicate filter criteria: log_2_ Fold change of ±1 and adjusted *p*-value of 0.05; blue: downregulated genes, red: upregulated genes; representative DEGs are specified. (D) Hierarchical clustering of all DEGs, based on Manhattan distances using Ward’s method. Data are presented as a heatmap normalized to the minimum and maximum of each neutrophil group. (E) Gene ontology (GO) analysis, showing the top 10 enriched GO categories for the significant clusters (C_1_, C_2_, and C_8_) from Figure 3D, using PANTHER 18.0. (F) GO analysis, showing all the angiogenesis pathways significantly enriched in cluster 2. Abbreviation: PC, Principal Component.

Next, we investigated whether these 2 waves of neutrophils were associated with different transcriptomic and functional profiles. We performed bulk RNA-seq analysis on FACS-sorted Ly6G^hi^Ly6C^int^CD11b^+^ neutrophils isolated from the liver at 24 hours and 72 hours post-CCl_4_. Since the number of intrahepatic neutrophils at 0 hours was very low, we used neutrophils isolated from the spleen of untreated mice as controls (Supplemental Figure S2, http://links.lww.com/HC9/A915). Principal component analysis clearly separated the liver neutrophil populations according to the time following injury (Figure 3A). Next, we performed a differential expression analysis to generate modules of genes that are significantly modulated in each neutrophil population (Supplemental Tables S4, S5, http://links.lww.com/HC9/A916, http://links.lww.com/HC9/A917). Using a cutoff of absolute fold change ≥0.5 and adjusted *p*<0.05, 1203 and 759 genes were differentially expressed in 24-hour and 72-hour neutrophils, respectively, as compared to control splenic neutrophils with 399 shared genes between the 2 time points (Figure 3B). There were 671 upregulated genes and 532 downregulated genes in 24-hour neutrophils, and 329 upregulated genes and 430 downregulated genes in 72-hour neutrophils as shown in the volcano plot (Figure 3C). Hierarchical clustering of differentially expressed genes (DEGs) showed distinct transcriptomic profiles of neutrophil subpopulations liver 24-hour, liver 72-hour, or spleen (control neutrophils) (Figure 3D). We identified eight distinct clusters. Gene ontology enrichment analysis revealed that cluster 1 genes, upregulated in both 24-hour and 72-hour neutrophils as compared to controls, were associated with the inflammatory response since they correspond to genes enriched in the “cellular response to TNF” and “response to wounding” pathways (Figure 3E). Cluster 2 genes that were only upregulated at 72 hours were significantly enriched in the “cellular response to TGF beta stimulus” and “regulation of angiogenesis” pathways (Figure 3E). Interestingly, only this cluster of genes was associated with a multitude of significantly enriched pathways related to angiogenesis (Figure 3F). Cluster 8 genes that were downregulated at 24 hours were primarily associated with metabolic processes and protein translation (Figure 3E). Clusters 3, 4, 5, 6, and 7 did not show any significantly enriched pathways, as proteins coded by genes of these clusters exhibited the least functional interactions with each other (Figure 3D).

These results were validated and confirmed by functional enrichment analysis of downregulated and upregulated DEGs, using over-representation analysis and gProfiler. This additional analysis demonstrated an inflammatory profile and altered metabolic and protein translation functions for 24-hour neutrophils, while uniquely 72-hour neutrophils exhibited pathways associated with tissue repair and angiogenesis (Supplemental Figures S3, http://links.lww.com/HC9/A915 and S4, http://links.lww.com/HC9/A915). Furthermore, protein-protein interaction network analysis of the DEGs identified 2 networks of highly interconnected nodes in 24-hour neutrophils, one associated with immune response and the other with protein translation (Supplemental Figure S5A, http://links.lww.com/HC9/A915). However, the analysis of DEGs from 72-hour neutrophils identified only one network associated with immune response (Supplemental Figure S5B, http://links.lww.com/HC9/A915). The comparison of the 2 immune response networks revealed the presence in 24-hour neutrophils of genes associated with: (1) noncanonical NF-κB pathway (upregulated genes) and (2) proteasome (downregulated genes). Furthermore, 72-hour neutrophils exhibited upregulation of the activating transcription factor 3 (*Atf3*) gene (Figure 3C and Supplemental Figure S5B, S6E, http://links.lww.com/HC9/A915), a stress-induced transcription factor that plays vital roles in anti-inflammatory immunity, oncogenesis, and tissue repair.^13^

To further validate these findings, we classified genes by functional categories and observed that 24-hour neutrophils exhibited several downregulated genes related to oxidative phosphorylation and protein translation (Supplemental Figure S6A, B, http://links.lww.com/HC9/A915). On the other hand, this neutrophil population demonstrated higher expression of genes associated with the NF-κB signaling pathway, especially *Relb* and *Nfkb2* (Supplemental Figure S6C, http://links.lww.com/HC9/A915). Furthermore, we identified 2 chemokine genes (*Ccl3* and *Ccl4*) with comparable expression in 24-hour and 72-hour neutrophils but higher than that of control splenic neutrophils (Supplemental Figure S6D, http://links.lww.com/HC9/A915). The proinflammatory signature of 24-hour neutrophils is underscored by overexpression of *Il12a* and to a lesser extent *Tnf* genes (encoding for the proinflammatory cytokines IL-12 and TNF, respectively). Intriguingly, we observed significantly high expression of *Il1b* gene encoding for IL-1β, a cytokine known for its angiogenic properties, in 72-hour neutrophils (Supplemental Figure S6D, http://links.lww.com/HC9/A915). These cells also exhibited expression of *Ptgs2* (*Cox-2*) or *Fgg* genes, further highlighting their angiogenic signature (Supplemental Figure S6E, http://links.lww.com/HC9/A915). Finally, we validated the results using quantitative RT-PCR comparing changes in the expression of 6 representative genes selected based on their functions—*Atf3*, *Fgg*, *Il1b*, *Ptgs2*, *Tnf*, and *Vegfa*. Expression patterns revealed by quantitative RT-PCR were comparable with RNA-seq results (Supplemental Figure S7, http://links.lww.com/HC9/A915).

### Gene Ontology: Biological Processing analysis based on GSEA reveals distinct enrichment of genes in 24-hour versus 72-hour neutrophils

**FIGURE 4:**
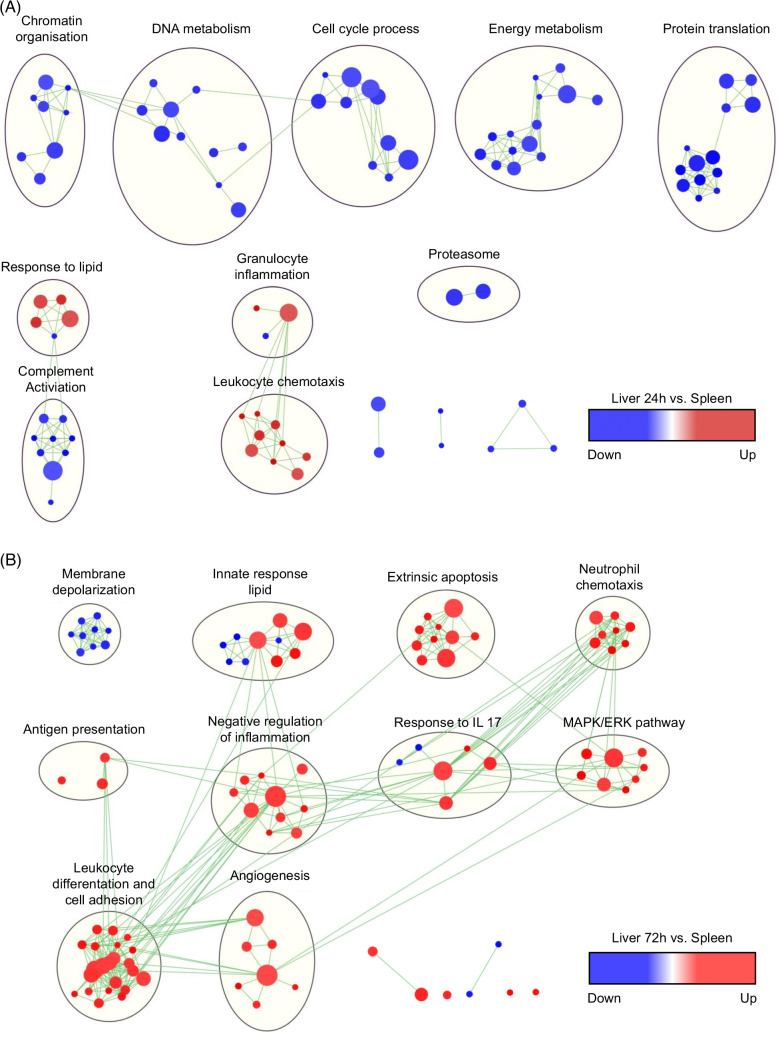
Gene set enrichment analysis demonstrates functionally distinct 24-hour and 72-hour postinjury liver neutrophils. (A) Enrichment maps of Liver 24-hour versus spleen and (B) liver 72-hour versus spleen. Gene set enrichment analysis was used to obtain enriched gene ontology terms in the biological process database, that were visualized using the Enrichment Map plug-in for Cytoscape (gene set size >10 genes, false discovery rate <0.2). Each node represents a gene ontology term, similar nodes were clustered together using the AutoAnnotate tool and connected by edges with the number of known interactors between the nodes being represented by the thickness of edges. Node color gradient represents the normalized enrichment scores. The size of each node denotes the gene set size for each specific node gene ontology term. Abbreviations: ERK, extracellular signal-regulated kinase; MAPK, mitogen-activated protein kinase.

To better dissect similarities and differences in the functional enrichment of gene sets in both neutrophil subsets as compared to control, we employed the Gene Ontology: Biological Processing analysis to evaluate a variety of biological responses. Based on the GSEA results, lists of functional gene sets (gene set size >10 genes; FDR<0.2) were generated. In total, 147 and 205 enriched pathways were detected for 24-hour and 72-hour liver neutrophils, respectively, as compared to controls (Supplemental Tables S6, S7, http://links.lww.com/HC9/A918, http://links.lww.com/HC9/A919). Enrichment maps were constructed to systematically visualize the enriched pathways as networks, thus allowing visual classification of gene sets from 24-hour neutrophils (Figure 4A) and 72-hour neutrophils (Figure 4B). Clusters associated with neutrophil chemotaxis and the response to lipids were enriched in both populations. On the other hand, the energy metabolism and protein translation gene sets were only enriched in 24-hour neutrophils, while angiogenesis and response to IL17 gene sets were only enriched in 72-hour neutrophils. These results are consistent with the data from the functional analysis based on DEG lists described above (Supplemental Figure S3, http://links.lww.com/HC9/A915 and S4, http://links.lww.com/HC9/A915).

In order to validate our observations obtained using spleen neutrophils as controls, we compared our data set to a recently published mouse neutrophil data set (GSE180824), containing 7 naive mouse liver neutrophil samples,^14^ hereinafter termed 0-hour neutrophils. DEG analysis confirmed the upregulation of the above-described genes, including *cxcr5* and *cxcr4* in 24-hour and 72-hour neutrophils, respectively (Supplemental Tables S8, S9, http://links.lww.com/HC9/A920, http://links.lww.com/HC9/A921). GSEA revealed similar pathways significantly enriched in 24-hour or 72-hour neutrophils when compared to 0-hour neutrophils as the ones described with the comparison to spleen neutrophils and ranking within the top 10 for this new analysis (Supplemental Figure S8, http://links.lww.com/HC9/A915). Notably, pathways such as “cytoplasmic translation” were negatively enriched in 24-hour versus 0-hour neutrophils (Supplemental Table S10, http://links.lww.com/HC9/A922). On the other hand, pathways like “response to IL-17,” “antigen processing and presentation,” and “cell migration involved in sprouting angiogenesis” were distinctively positive in the 72-hour versus 0-hour neutrophils (Supplemental Table S11, http://links.lww.com/HC9/A923).

In summary, 24-hour neutrophils exhibited an inflammatory profile with the upregulation of genes and pathways associated with response to wounding, inflammatory cytokines, and the noncanonical NF-κB pathway while the 72-hour neutrophils exhibited an angiogenic profile with the upregulation of genes associated with angiogenesis and tissue repair.

### 24-hour neutrophils show reduced protein translation

**FIGURE 5:**
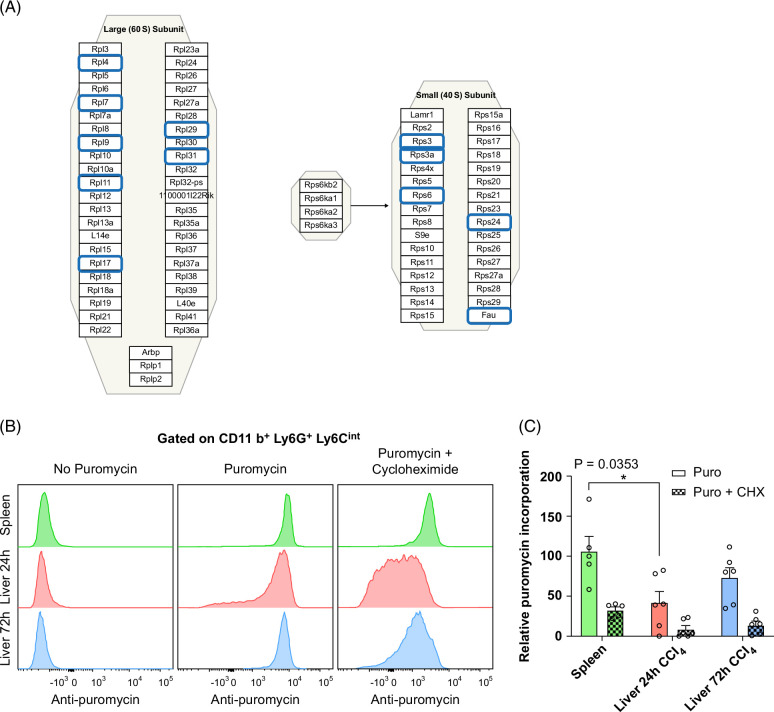
Neutrophils from 24 hours after injury exhibit a reduction in protein translation. (A) Cytoplasmic ribosomal proteins involved in translation for *Mus musculus from Wikipathways.* Ribosomal protein genes boxed in blue represent significant downregulated DEGs in the comparison Liver 24-hour versus Spleen. https://www.wikipathways.org/pathways/WP163.html. (B) Representative histograms for isolated neutrophil populations treated with puromycin (10 µg/mL) or puromycin (10 µg/mL) + CHX (100 µg/mL). (C) Relative puromycin incorporation from gMFI normalized on the control condition (spleen). N = 5–6 mice per group. Data are shown as mean±SEM. Statistical analysis was performed using one-way ANOVA on ranks and Dunn’s Multiple Comparison Test. *p<0.05. Abbreviations: CCl_4_, carbon tetrachloride; CHX, cycloheximide.

We observed the downregulation of several genes involved in protein translation in 24-hour neutrophils. These genes encode ribosomal proteins and translation initiators, such as *Eif5* (Figure 5A, Supplemental Figures S5A and S6B, http://links.lww.com/HC9/A915, http://links.lww.com/HC9/A918). For functional validation, we performed a puromycin incorporation assay to quantify newly synthesized proteins. Upon treatment with puromycin (Figure 5B, C), we observed significantly lower labeling in 24-hour neutrophils (*p* = 0.0353), reflecting a lower number of nascent polypeptides as compared to control splenic neutrophils. There was a trend of reduced translational capacity in 72-hour neutrophils as well, but results were not statistically significant when compared to control. In the presence of puromycin + cycloheximide (an inhibitor of eukaryotic translation elongation) used as a negative control, we observed repression of protein translation in the 3 neutrophil groups tested. These results confirm and validate, at the functional level, the reduction of protein translation in 24-hour neutrophils.

### Altered mitochondrial metabolism in 24-hour neutrophils

**FIGURE 6:**
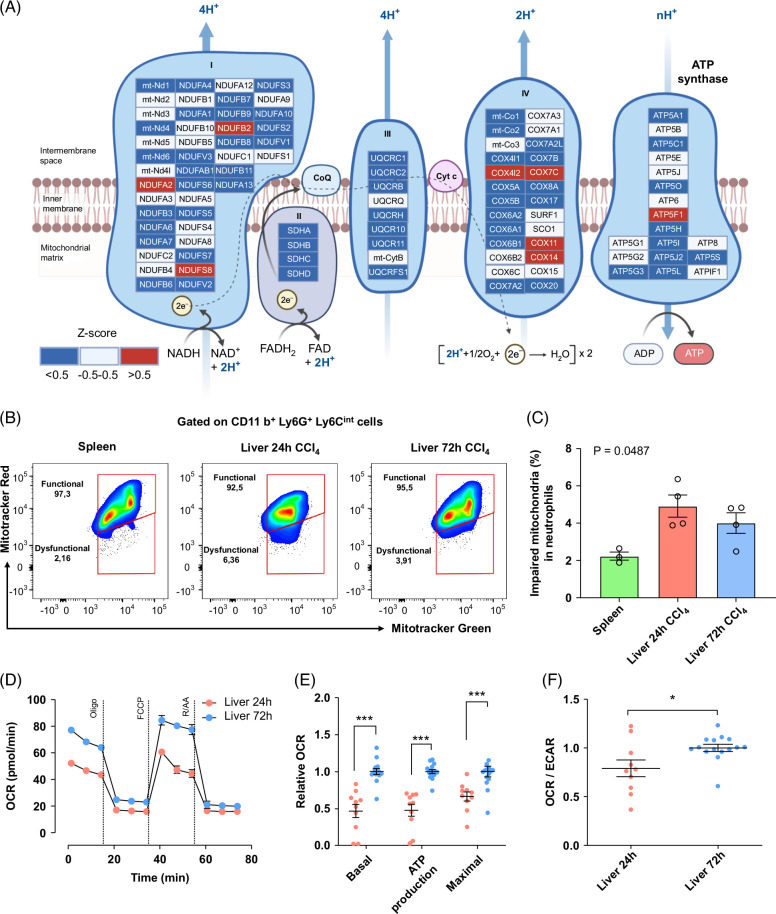
Neutrophils from 24 hours after injury exhibit a reduction in mitochondrial functionality. (A) Electron transport chain activity of 24-hour neutrophils based on mRNA expression. The relative gene expression levels of electron transport chain were calculated by Z score based on the gene expression of all samples included in the study. Colored nodes represent gene expression Z score in early neutrophils (blue = low, gray = no change, and red = high). The edges represent different types of protein interactions. (B) Representative density plots of mitochondrial membrane potential in neutrophils stained with the mitochondrial probes Mitotracker Green and Mitotracker Red analyzed by flow cytometry. (C) Quantification of the percentage of dysfunctional mitochondria in the different neutrophil subtypes. N = 3–4 mice per group. Data are shown as mean±SEM. Statistical analysis was performed using One-way ANOVA on ranks and Dunn Multiple Comparison Test. *p<0.05. (D) Representative mitochondrial stress test in 24-hour and 72-hour liver neutrophils (oligomycin 2  µM, FCCP 0.5  µM, and Rot/AA 0.5 µM) analyzed by an XFe96 Extracellular Flux Analyzer. (E) Mitochondrial basal and maximal and ATP-linked respiration are quantified from 3 independent experiments. N = 12 mice per group. Data are shown as mean±SEM. Statistical analysis was performed using Mann-Whitney *U* test. ***p<0.001 (F) OCR/ECAR ratios of 24-hour and 72-hour neutrophils. Data were analyzed by Mann-Whitney *U* test. *p<0.05. Abbreviations: CCl_4_, carbon tetrachloride; COX, cyclooxygenase; ECAR, extracellular acidification rate; FAD, flavin adenin dinucleotide; FADH_2_, dihydroflavin adenin dinucleotide; FCCP, carbonyl cyanide-4-(trifluoromethoxy) phenylhydrazone; OCR, oxygen consumption rate.

We observed an overall reduction in energy metabolism and mitochondrial respiration in 24-hour neutrophils (Figure 4A, Supplemental Figure S6A, http://links.lww.com/HC9/A918). We also observed reduced expression of genes involved in the electron transport chain and oxidative phosphorylation (Figure 6A), and thus energy production. This finding suggested that 24-hour neutrophils shifted their metabolism from a high energy–demanding status using mitochondrial respiration to a glycolytic-based rapid ATP production. To validate this hypothesis, we investigated the neutrophil mitochondrial function using flow cytometry staining based on mitochondrial mass (MitoTracker Green probe) and mitochondrial membrane potential (ΔΨm; MitoTracker Red probe). We gated on live 24-hour and 72-hour neutrophils (CD11b^+^Ly6G^+^Ly6C^int^) where mitochondria were considered functional in MitoTracker Green^high^/MitoTracker Red^high^ cells and dysfunctional in MitoTracker Green^high^/Mitotracker Red^low^ ones. Compared to other populations, 24-hour neutrophils exhibited a higher proportion of cells with dysfunctional mitochondria (*p* = 0.0487) (Figure 6B, C). Furthermore, the Mito Stress Test performed on FACS-sorted neutrophils demonstrated significantly lower basal and maximal mitochondrial oxygen consumption rate in 24-hour neutrophils as compared to 72-hour neutrophils (Figure 6D, E). Finally, analysis of extracellular acidification rates (ECAR), a measurement related to the production of lactic acid and therefore the glycolytic rate, revealed that oxygen consumption rate/ECAR ratios of 24-hour neutrophils were lower than those of 72-hour neutrophils (Figure 6F), suggesting a lower propensity for mitochondrial function as opposed to glycolysis to meet energy demands. In summary, 24-hour neutrophils exhibited altered mitochondrial metabolism with a shift toward glycolysis.

### 24-hour neutrophils exhibit high ROS production

**FIGURE 7:**
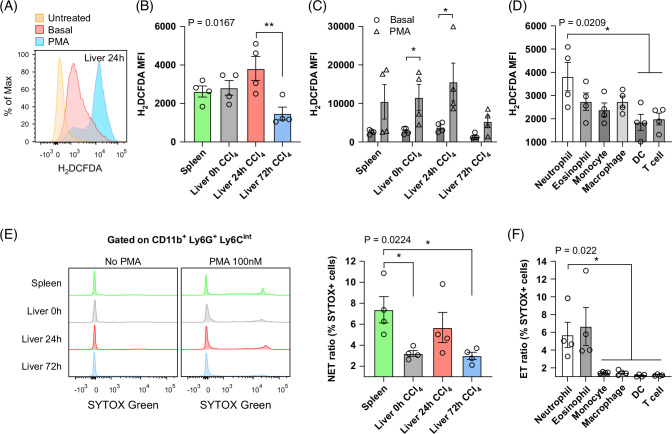
Neutrophils from 24 hours after injury produce high levels of reactive oxygen species (ROS) and are prone to NETosis. (A) Representative flow cytometry analysis of CM-H2DCFDA (5 µM) staining for ROS in 24-hour neutrophils post-carbon tetrachloride (CCl_4_) injury treated or not with PMA (50 nM). (B) Flow cytometry analysis of CM-H2DCFDA staining for baseline ROS in neutrophil populations. (C) Flow cytometry analysis of CM-H2DCFDA staining for ROS in neutrophil populations treated with or without PMA. Statistical analysis was performed using Mann-Whitney test. *p<0.05. (D) Flow cytometry analysis of CM-H2DCFDA staining for baseline ROS in intrahepatic leukocytes at 24 hours post-CCl_4_ injury. N = 4 mice per group. Data are shown as mean±SEM. Statistical analysis was performed using one-way ANOVA and Dunn Multiple Comparison Test. *p<0.05, **p<0.01. (E) Representative histograms (left) and NET ratio (right) from SYTOX green+ neutrophils isolated from spleen, liver 0 hours, 24 hours, and 72 hours post-CCl4 injury treated with or without PMA 100 nM and analyzed by flow cytometry. The NET ratio is calculated as induction of NETosis: (% SYTOX Green+ PMA/% of control). (F) ETs induction in intrahepatic leucocytes at 24 hours post-CCl_4_ injury. N = 4 mice per group. Data are shown as mean±SEM. Statistical analysis was performed using one-way ANOVA and Dunn Multiple Comparison Test. *p<0.05, **p<0.01. Abbreviations: DC, dendritic cell; ET, extracellular traps; H_2_DCFDA, 2′,7′-Dichlorodihydrofluorescein diacetate; MFI, mean fluorescence intensity; NET, neutrophil extracellular trap; PMA, phorbol 12-myristate 13-acetate.

Recruitment of neutrophils to sites of injury and their inflammatory properties correlate with increased ROS production. ROS disrupts cellular homeostasis via altering mitochondrial functions and lipid, protein, and nucleic metabolism while sustaining proinflammatory signals.^15^ We quantified intracellular ROS production using the fluorescent probe CM-2′,7′-Dichlorodihydrofluorescein diacetate. A representative staining is presented in Figure 7A. 24-hour neutrophils were the main ROS-producing population (Figure 7B). Intracellular ROS was significantly reduced in 72-hour neutrophils during repair. In the presence of phorbol 12-myristate 13-acetate, used as a positive control, all neutrophil populations exhibited increased ROS production (Figure 7A, C). To further confirm the specificity of our results, we evaluated ROS in all intrahepatic leukocytes. ROS was produced primarily by myeloid cells, predominantly neutrophils (Figure 7D). These observations demonstrate that 24-hour neutrophils are significant producers of ROS, concomitant with their assigned signature as proinflammatory neutrophils.

### 24-hour neutrophils are prone to phorbol 12-myristate 13-acetate-induced NETosis

Because activated neutrophils can potently produce neutrophil extracellular traps (NETs) that are associated with poor outcomes in patients with ALI,^16^ we examined the capacity of the neutrophil populations to undergo NETosis *in vitro* upon phorbol 12-myristate 13-acetate stimulation using SYTOX green. Liver 24-hour neutrophils exhibited the highest capacity to generate NETs (Figure 7E). This level of NETs was comparable to spleen neutrophils, known for their efficient NETosis.^17^ To confirm the specificity of our assay, we quantified extracellular traps in other populations of intrahepatic leukocytes or spleen. As expected, only hepatic neutrophils and eosinophils were able to generate extracellular traps^18^ (Figure 7F).

### Distinct CXCLs/CXCRs signaling axes in 24-hour versus 72-hour neutrophils

Next, we investigated the liver gene expression of several neutrophil-recruiting chemokines and their respective receptors on the neutrophil surface. First, we examined the expression of *Cxcr2*, the key receptor usually implicated in neutrophil migration and recruitment. It was strongly expressed; however, there was no difference in the expression levels between 24-hour, 72-hour, and control neutrophil populations (Supplemental Figure S6F, http://links.lww.com/HC9/A915). We next quantified the intrahepatic gene expression of three C-X-C chemokine receptor 2 (CXCR2) ligands: *Cxcl1*, *Cxcl2*, and *Cxcl5*. We observed an upregulation of *Cxcl1* and *Cxcl2* at 12 hours right before the recruitment of the first wave (24 hours) of neutrophils during necroinflammation (Supplemental Figure S9A, B, http://links.lww.com/HC9/A915). Interestingly, these chemoattractants are known for their recruitment of pathological neutrophils associated with liver damage and are targets of therapeutic strategies.^19,20^ On the other hand, the second wave (72 hours) of neutrophils was temporally associated with increased expression of *Cxcl5* and almost baseline levels of *Cxcl1* and *Cxcl2* (Supplemental Figure S9C, http://links.lww.com/HC9/A915).

**FIGURE 8:**
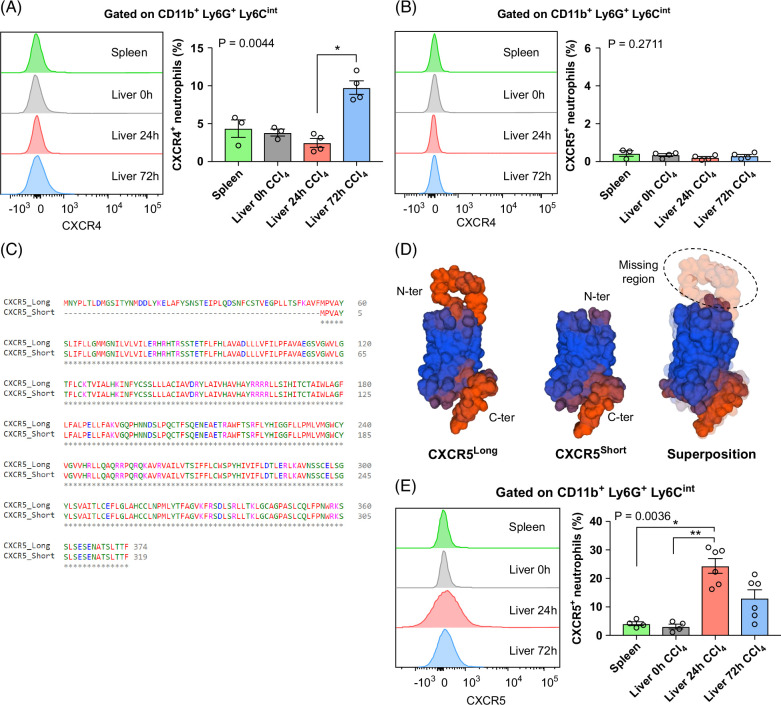
CXCR4 and CXCR5 markers distinguish recruitment signals of 24-hour versus 72-hour postinjury liver neutrophils. (A) Representative histograms and frequencies of CXCR4^+^ neutrophils isolated from spleen, liver 0 hours, 24 hours, and 72 hours post-CCl_4_. N = 4 mice per group. (B) Representative histograms and frequencies of CXCR5^+^ neutrophils using clone 2G8 (N-ter) isolated from spleen, liver 0 hours, 24 hours, and 72 hours post-CCl_4_. N = 4 mice per group. (C) Multiple sequence alignment using Clustal Omega (ClustalO) presenting the aligned sequences of short and long CXCR5 isoforms. (D) Predicted structure of short and long CXCR5 isoforms and their superposition. The local quality estimation with a chart for target by AlphaFold Model (SWISS-Modeler) is represented in blue as high confidence degree and in red as lower. (E) Representative histograms and frequencies of CXCR5^+^ neutrophils using clone C-3 (C-ter) isolated from spleen, liver 0 hours, 24 hours, and 72 hours post-CCl_4_. N = 6 mice per group. Data are shown as mean±SEM. Statistical analysis was performed using one-way ANOVA and Dunn Multiple Comparison Test. *p<0.05, **p<0.01. Abbreviations: CCl_4_, carbon tetrachloride; CXCR, C-X-C chemokine receptor.

In search of markers that can discriminate between 24-hour and 72-hour neutrophils, we examined gene expression of immune surface receptors that were differentially expressed in these 2 populations (Supplemental Figure S6F, http://links.lww.com/HC9/A915). We observed strong expression of *Cxcr5* in 24-hour neutrophils (Figure 3C, D, Supplemental Figure S6F, http://links.lww.com/HC9/A915). In contrast, 72-hour neutrophils exhibited upregulation of *Cxcr4*, recognized as a neutrophil marker with angiogenic properties.^21,22^ To validate the use of these two receptors as phenotypic markers, we examined the surface expression of CXCR4 and CXCR5 on 24-hour and 72-hour neutrophils by flow cytometry. We observed an increased expression of CXCR4 on 72-hour neutrophils as compared to 24-hour neutrophils (*p* = 0.0044) (Figure 8A). Although CXCR4^+^ neutrophils were mainly detectable at 72 hours post-CCl_4_, they only represented about 10% of the total neutrophils at this time point (Figure 8A). In contrast, we could barely detect CXCR5 on the surface of neutrophils from the different time points (0.35%–0.55%, *p* = 0.27) using an anti-mouse CXCR5 antibody targeting the N-terminus of the receptor (Figure 8B), which was in contradiction with our transcriptomic analysis (Supplemental Figure S6F, http://links.lww.com/HC9/A915). To determine if the inability to detect surface expression of CXCR5 was due to the expression of a different isoform, we extracted the *Cxcr5* sequence expressed by neutrophils from our RNA-seq data and aligned it to the *Cxcr5* sequence from NCBI (NCBI Reference Sequence: NP_031577.2). We found a deletion of 165 nucleotides in the N-terminal region as compared to the *Cxcr5* sequence from NCBI corresponding to a protein that is 55 aa shorter (Figure 8C, D). The presence of a short isoform of the CXCR5 protein was previously reported on human blood monocytes, but not in mice. We then used another anti-CXCR5 antibody targeting the conserved C-terminal region present in both isoforms and observed significantly increased expression of CXCR5 on 24-hour neutrophils as compared to control liver (0 hour) or spleen (*p* = 0.0036) (Figure 8E).

To determine if this differential expression of chemokine receptors is linked to different chemokine signals, we evaluated the hepatic expression of genes encoding the chemokines CXCL13 and CXCL12, ligands of CXCR5 and CXCR4, respectively. *Cxcl13* peaked at 12 hours post-CCl_4_ (Supplemental Figure S9D, http://links.lww.com/HC9/A915), just before the early peak of neutrophils at 24 hours. In contrast, we observed a constant level of expression of *Cxcl12* up to 72 hours after injury when this expression dropped (Supplemental Figure S9E, http://links.lww.com/HC9/A915) coincident with the late neutrophil peak.

These findings demonstrate the differential regulation of the 2 waves of neutrophils at the chemokine level and support a different phenotypic profile for each population with preferential expression of a short isoform of CXCR5 on 24-hour neutrophils.

## DISCUSSION

In this study, we identified 2 waves of neutrophils infiltrating the liver during the wound healing response to ALI at 24 hours and 72 hours, respectively. These 2 neutrophil populations exhibited distinct transcriptomic and functional profiles. The 24-hour neutrophils were proinflammatory and the 72-hour neutrophils were prorepair.

Early 24-hour neutrophils present a proinflammatory profile as demonstrated by the upregulation of genes associated with known signatures of neutrophils in early liver injury such as the noncanonical NF-κB pathway and inflammatory cytokines such as IL12a.^23^ They also exhibited reduced protein translation and mitochondrial dysfunction consistent with their lower energy status, decreased expression of oxidative phosphorylation genes, and dysfunctional mitochondria. Indeed, differences in the activation states of immune cells dictate the metabolic machinery they use for energy production.^24^ Upon activation, immune cells require faster ATP use to sustain the production of inflammatory mediators and would thus shift to glycolysis as their energy source.

The 72-hour neutrophils exhibited a prorepair profile consistent with the findings in the thermal sterile injury model, whereby depleting neutrophils resulted in larger lesions, lower collagen deposition, delayed revascularization, and abrogated healing.^7^ The resolving signature of 72-hour neutrophils is underscored by the upregulation of the *Atf3* and *Ptgs2* genes. *Atf3* codes for the transcription factor ATF3, known for its suppressive functions in innate immune cells, modulation of proinflammatory gene expression,^25,26^ and regulation of macrophage responses.^13^ *Ptgs2* codes for cyclooxygenase-2, responsible for the conversion of arachidonic acid to prostaglandin H2 and is implicated in M1/M2 polarization and angiogenesis.^27–29^

We identified 2 surface markers that differentiate the 2 neutrophil populations. The chemokine receptor, CXCR4, was enriched on the 72-hour prorepair neutrophils. This is in line with the association of CXCR4 expression in neutrophils with angiogenesis and tissue repair.^30,31^ Using a model of neutrophil fate tracking in different tissues, Ballesteros et al^32^ identified various neutrophil states with different noncanonical functions, including vascular repair. The depletion of neutrophils in this model compromised angiogenesis.^32^ These functions were CXCR4-dependent and occurred in CXCL12-rich areas in tissues. Moreover, the CXCR4/CXCL12 receptor/ligand pair regulates the trafficking of neutrophils.^33^ Release of mature neutrophils from bone marrow is mediated by attenuation of CXCR4 signaling while re-expression of CXCR4 on neutrophils promotes the return of “aged” neutrophils to the bone marrow for clearance.^34^ Finally, in the thermal injury model, neutrophils penetrated the injury site and dismantled injured vessels while enhancing the regrowth of new ones, then re-entered the vasculature in a process termed reverse migration toward the bone marrow. Few of these transmigrating neutrophils pass by the lungs where they upregulate CXCR4 before entering the bone marrow in a CXCL12-dependent manner to undergo apoptosis.^7,35^ Interestingly, we observed decreased expression of CXCL12 in the liver after 72 hours, suggesting a mechanism to override the retention of CXCR4^+^ neutrophils and allowing their reverse migration into the circulation.

The 24-hour inflammatory mouse neutrophils expressed a short isoform of CXCR5. A similar isoform was previously described on human blood monocytes.^36^ Different chemokine receptor isoforms can lead to differential immune cell recruitment and activation.^37,38^ Limited studies have examined the role of CXCR5 on myeloid cells. CXCR5 can promote myeloid-derived suppressor cells migration toward malignant tissues and their accumulation.^39^ CXCR5 also contributed to the differentiation of the promyeloblastic cell line HL-60 into neutrophil-like cells.^40^ Additional studies are needed to understand the role of these different isoforms in neutrophil function.

It is difficult to assess whether these 2 waves of neutrophils represent different recruitment events or differentiation in situ. We cannot exclude the possibility of neutrophil differentiation within the liver in response to different stimuli during various phases of the wound healing response. However, the short half-life of neutrophils^32^ and their decline at 48 hours suggest 2 distinct recruitment events. Furthermore, several chemokines are implicated in the recruitment of neutrophils into the injured liver.^41^ The expression of different surface receptors as well as different chemokines prior to the 24-hour versus the 72-hour wave of neutrophils suggest that they are recruited in response to different signals.

Although our findings suggest heterogeneity of neutrophils during the different stages of injury and repair, the prevalence of a particular neutrophil subset at a specific phase cannot be confidently resolved by the bulk phenotyping and RNA-seq approaches we used. The isolation methods we undertook, although widely used, may have affected our capacity to detect more fragile or rare neutrophil populations. Future studies using single-cell–based approaches may allow a better assessment of the heterogeneity and trajectories of all neutrophil subpopulations.

Neutrophil heterogeneity was also reported in other models of ALI. Two neutrophil subpopulations were observed in the liver during acetaminophen-induced or thioacetamide-induced ALI, where CXCL2^+^ neutrophils exhibited a proinflammatory signature and were implicated in disease progression.^42^ Another study demonstrated that Ly6C^hi^ monocytes induce transcriptional changes in neutrophils that are associated with increased inflammatory phenotype and activity like ROS production.^43^ This inflammatory transcriptional profile was associated with proteolysis and negative regulation of metabolism,^43^ consistent with our observation of the combined inflammatory/low protein translation/low metabolic activity signature of 24-hour neutrophils. Concomitantly, the release of ROS by neutrophils promoted the conversion of proinflammatory monocytes/macrophages to proresolving macrophages, contributing to liver repair.^8,43^ Our data support this concept of intercellular cooperation among myeloid cells and with other cells such as HSCs to orchestrate tissue repair. Future studies using spatial transcriptomics can better elucidate these interactions and their timings.^44^ Finally, it would be relevant to assess neutrophil heterogeneity during chronic liver injury as it may play a beneficial role in the resolution of liver inflammation and fibrosis.^3^

Given the challenges of studying ALI in humans, the characterization of neutrophils in mouse models provides valuable mechanistic insights.^45^ Activated neutrophils were detected in the peripheral blood of patients with acetaminophen-induced ALI, but hepatic neutrophils could not be examined.^45,46^ Transcriptomic studies demonstrated intrahepatic neutrophil heterogeneity in humans, but mouse models were essential for functional studies.^47,48^ In accordance with our findings, subjects with severe alcoholic hepatitis exhibited 2 different histological phenotypes with high and low hepatic neutrophil infiltration where high neutrophils correlated with liver inflammation and disease progression.^48^ Studies in mouse models demonstrated ROS-mediated inflammatory signature of hepatic neutrophils during the binge-alcohol phase (acute stimuli to the liver),^48^ and resolving neutrophils were involved in tissue repair via miR-223.^48,49^

In conclusion, by investigating the recruitment kinetics, transcriptomic profile, and functions of different neutrophil subsets during ALI, we identified 2 populations that play distinct roles as proinflammatory or prorepair. These findings may guide the development of new interventions modulating neutrophil recruitment to the liver and improving outcomes of ALI.

## Supplementary Material

SUPPLEMENTARY MATERIAL
