# Supplementary material for: Two transcriptionally and functionally distinct waves of neutrophils during mouse acute liver injury
Source: Hepatol Commun. 2024 Jun 19;8(7):e0459. doi: 10.1097/HC9.0000000000000459 (PMC11186811; doi:10.1097/HC9.0000000000000459)
Supplement: SUPPLEMENTARY MATERIAL [file hc9-8-e0459-s001.pdf]

Supplementary Materials

Two Transcriptionally and Functionally Distinct Waves of Neutrophils during Mouse Acute Liver Injury

Yousef Maali[1,2], Manuel Flores Molina[1,2], Omar Khedr[1], Mohamed N. Abdelnabi[1,2], Jessica Dion[1], Ghada S. Hassan[1], Naglaa H. Shoukry[1, 3,*]

[1]Centre de Recherche du Centre hospitalier de l'Université de Montréal (CRCHUM), Montréal, QC, Canada.

[2]Département de microbiologie, infectiologie et immunologie, Université de Montréal, Montréal, QC, Canada.

[3]Departement de médecine, Université de Montréal, Montréal, QC, Canada.

Correspondence to: naglaa.shoukry@umontreal.ca

This PDF file includes:

Extended Materials and Methods

Figs. S1 to S9

Table S1 and S3

Legends for Tables S4 to S11

Other Supplementary Material for this manuscript include the following:

Tables S4 to S11

Extended Materials and Methods

Isolation of intrahepatic neutrophils

Liver neutrophils were isolated from hepatic tissue through a two-step process, involving mechanical dissociation followed by enzymatic digestion. Initially, the liver was finely minced using a scalpel. The resulting dissociated tissue was suspended in cold RPMI media supplemented with 10% FBS, benzonase (Millipore Sigma, Burlington. MA, USA; 0.2 U/mL), and collagenase D (Roche, Basel, Switzerland; 0.1 mg/mL). Subsequently, the suspension was incubated at 37°C with rotational agitation for a duration of only 20 minutes. The tissue was further mechanically dissociated by passing it through a 70 µm cell strainer. The cell suspensions were subjected to 37% isotonic Percoll gradient (Millipore Sigma) centrifugation to separate RBCs and leukocytes from hepatic cells. The resulting cells were hemolyzed with ACK lysing buffer solution (Gibco) to obtain intrahepatic leukocytes (IHLs). Neutrophils were purified from liver leukocytes using the Anti-Ly-6G MicroBeads UltraPure, mouse kit (Miltenyi-Biotec, Bergisch Gladbach, Germany) following the manufacturer's instructions. The purity of the neutrophils was assessed by flow cytometry upon staining of the cell suspensions with anti-Ly6G+6C antibodies and CD11b (1).

Flow cytometry

To detect surface markers, freshly isolated neutrophils were first washed with FACS buffer (PBS, 1% FBS, and 0.02% Sodium Azide). Subsequently, they were transferred to a 96-well plate and incubated in the dark at 4°C for 30 minutes with a combination of fluorescently conjugated antibodies along with the viability dye aqua vivid (Thermofisher). Samples were then washed and stored at 4°C in the dark until acquisition, which took place on the same day. For detection of live cells, the Aqua Live/Dead Fixable Dead Cell Stain kit was used (Life Technologies, Burlington, Ontario, Canada). The acquisition was performed using a BD LSRFortessa™ Cytometer equipped with violet (405 nm), blue (488 nm), yellow-green (561 nm), and red (633 nm) lasers, utilizing FACSDiva version 8.0.1 (BD Biosciences, San Diego, CA). The analysis of the acquired data was carried out using FlowJo version 10.4 for Mac (BD Biosciences). Please refer to Table S1 for detailed information regarding the antibodies used in the flow cytometry analysis.

Immunofluorescence

Immunofluorescence (IF) for MPO was performed on 4 mm FFPE tissue sections as previously described (2). Samples were stained with the primary antibody Goat anti-mouse MPO (R&D Systems, MN, USA). Donkey anti-goat secondary antibody conjugated to Alexa fluor 568 (Invitrogen, MA, USA) was used for the revelation. Whole tissue images were acquired using the whole slide scanner Olympus BX61VS. To address any potential issues related to autofluorescence, our approach involved selecting IF channels with minimal autofluorescence in the liver or those with minimal spill-over. Furthermore, we utilized the VIS software (Visiopharm, Westminster, CO) for quantification purposes, which facilitated the determination of positivity thresholds for each individual channel. The pixel intensity values were set considerably higher than the background signal, and any visual artifacts present in the analyzed regions were eliminated. To ensure the absence of nonspecific binding of primary antibodies, we employed specific staining patterns tailored to the tissue or cellular location of the target marker (e.g., nuclear, cytoplasmic, around the CV area, etc.). We confirmed the specificity of the staining by utilizing other antibodies derived from the same species as negative controls. Multiplex immunofluorescence (mIF) for IBA1, αSMA, Desmin and MPO was done on frozen 5μm OCT-embedded and fixed liver sections as previously described (3). All antibodies used are listed in Supplementary Table S1.

Image analysis

Image analysis was executed utilizing the VIS image analysis software. The Author™ module within VIS was employed to design the subsequent protocols: 1) Automated identification and quantification of the tissue area; 2) Identification and quantification of the MPO^{+} area. For the automated alignment of images obtained from serial sections, the Tissuealign module of VIS was utilized. Heatmaps depicting MPO^{+} cells were generated using the tissue heatmap function of VIS. Nuclei detection was performed employing the protocol 10169 - Nuclei Detection, AI (Fluorescence) from Visiopharm.

RNA isolation and RT-PCR analysis

Total RNA was isolated from murine hepatic tissue using RNeasy Mini Kit (Qiagen, Hilden, Germany). cDNA was generated from 2 μg of total RNA using the Transcriptor Universal cDNA Master Mix (Roche Life Science, Penzberg, Germany). cDNA was amplified using the LightCycler® 480 SYBR Green I Master (Roche) in the LightCycler 480 instrument (Roche). All the previous procedures were performed according to the manufacturer's

protocols. mRNA expression was normalized to the expression of the housekeeping gene 28s and was determined using the 2-ΔΔCt method. For neutrophils, RT-PCR was performed on RNA from neutrophils FACS sorted. The RNA was isolated using the RNeasy micro-kit (Qiagen). cDNA was generated from 300 ng of total RNA. The rest of the process was conducted as above. Primer sequences are listed in Table S2**.**

Differential gene expression and functional analysis

Prior to normalization using the trimmed mean of M values (TMM) method from the edgeR package (4), genes with low expression values were removed. Only genes with more than one read count per million (cpm) in at least three samples were included in the following analysis). Principal component analysis (PCA) was performed on the normalized data and visualized using the ggbiplot library.

Differential expression analysis was performed using the voom function (5) of the limma package (6). The resulting P values were adjusted using Benjamini and Hochberg's approach for controlling the false discovery rate (FDR). Genes with an adjusted P-value <0.05 found by voom/limma were assigned as differentially expressed genes (DEG's). Using ward.D method, a hierarchical clustering heatmap was generated presenting the log2(FPKM+1) of DEG union within all comparison groups (7). Volcano plots visualization was performed using the online platform SRplot (http://www.bioinformatics.com.cn/srplot). The DEG's lists obtained were entered into NetworkAnalyst 3.0 platform (https://www.networkanalyst.ca/NetworkAnalyst/) to construct Protein-protein interaction (PPI) networks (8). The DEG's were displayed together in a list with the official gene symbol, along with the expressions as fold change. The STRING Interactome was selected as the PPI database that is with medium (400) - high (1000) confidence score. The confidence score cut-off was set at 900 for the analysis. The seeds were mapped to the corresponding molecular interaction database and the subnetworks with at least 10 nodes are demonstrated. The degree of each node was calculated based on the number of its connections to other nodes. In the network, the color of a certain node indicates its expression while the area represents the degree of it.

Functional enrichment analysis of the up/down-regulated gene cluster was performed using g:GOSt function in g:Profiler (9). The selected organism was *Mus musculus*, the significance threshold was g:SCS, with a threshold of 0.05. Gene Ontology (GO) databases were inquired. GO enrichment analysis powered by PANTHER 18.0 was performed directly from the home page of the GO website (https://geneontology.org/) on

all the genes clusters generated by the hierarchical clustering (10). Only GO terms of biological process data base with FDR $P < 0.05$ (Fisher's exact test), were considered as significant.

The lists of pre-ranked genes given by LIMMA moderated t-statistic were used to perform gene set enrichment analysis (GSEA), using the fgsea function from the fgsea package (11). The GO, Biological Process (BP) database was used as gene sets for GSEA. Visualisation of significantly enriched GO terms of biological process between subtypes was done in the Cytoscape plugin, Enrichment Map (12). Automatic annotation of the clusters networks was conducted using AutoAnnotate Cytoscape application.

Publicly available RNAseq datasets

Gene sets derived from RNAseq analysis of naïve liver neutrophils (from GSM5471589 to GSM5471595) were extracted from the GSE180824 datasets. To effectively analyze data and adjust for batch effects, an additional batch variable was integrated into the linear model, using the same voom/limma workflow. Control Ly6G^{high} neutrophil samples (from GSM5471596 to GSM5471602) have been included in the analysis to normalize the data between the two studies. Contrasts between 24h and 72h liver neutrophils, were compared to naïve liver neutrophils (from GSM5471589 to GSM5471595) as described above.

In silico analysis of the CXCR5 sequence

From our sequencing data, the consensus sequence of the CXCR5 was extracted by subsetting the aligned BAM files to contain only the short reads within the same chromosome and position as the CXCR5 gene - using SAMtools (13) . After which, a series of contig sequences were generated from these short reads using the Spades command line software (14). Using the alignment software seaview (15) , the generated contig sequences were aligned to known CXCR5 sequence from NCBI (transcript_id=XM_036154589.1), in order to identify the relevant section contig containing the CXCR5 sequence expressed by neutrophils. Tertiary structural details of the new CXCR5 predicted protein were analyzed using the online software SWISSMODEL (16) . The structures of predicted CXCR5 protein were constructed by molecular modeling, using the AlphaFold DB model of CXCR5_RAT (P34997.1.A). The sequence identity between target and template is 96.87%. The GMQE (Global Model Quality Estimation) is 0.85.

Through the Swiss model, a comparison of the tertiary structure model of the short and long CXCR5 isoforms were conducted using the tool structure comparison.

Mitochondrial functionality assay

Freshly isolated neutrophils (1.5×10^5 cells) were seeded per well in 96 round bottom well plates. Cells were resuspended in pre-warmed antibody and probes mix in PBS + 2% FBS for 15 min at 37 °C. Cells were then incubated at 37 °C for 15 min in PBS +2% FBS with a mixture of 50nM MitoTracker Green FM (Thermo Fisher Scientific, Waltham, MA, USA), 50nM MitoTracker Red CMXRos (Thermo Fisher Scientific) and phenotyping antibodies. At the end of incubation, samples were washed twice with pre-warmed PBS + 2% FBS, and directly acquired in the flow cytometer (17).

Puromycin incorporation assay

We performed all experiments in 96 wells round bottom plates, with 1.5×10^5 cells/well in 150 µl RPMI complete medium including 10 % FBS. Additional wells for each isolated neutrophil time points were added as controls: (i) no Puromycin (Millipore Sigma) and (ii) Puromycin treated with the translation inhibitor Cycloheximide (CHX; Millipore Sigma). For CHX-treated control wells, CHX was previously added to complete medium at a final concentration of 100 µg/mL, for 30 min at 37°C. Media was aspirated and pre-warmed cell culture medium was added to no-Puromycin control wells, medium supplemented with Puromycin (10 µg/mL) and CHX (100 µg/mL) to CHX-treated control wells, and medium supplemented with Puromycin (10 µg/mL) to the main experimental wells. Cells were incubated for 30 min at 37°C. Cells were centrifuged at room temperature (RT), two times washed with 150 µl prewarmed medium, and stained for surface and viability marker. After 30 min incubation at 4°C, the cells were washed twice and incubated with permeabilization/ fixation buffer (BD Cytofix/Cytoperm™) for 30 minutes at 4°C in the dark. Next, samples were washed and incubated for 30 min at 4°C in the dark with the Anti-Puromycin antibody, Alexa Fluor™ 647 Conjugate. Finally, samples were washed and kept at 4°C in the dark up to acquisition time.

Real time bioenergetic profile analysis

Mito Stress Test was performed using the Seahorse XFe96 Analyser (Agilent Technologies, Santa Clara, CA, USA) to measure oxygen consumption rate (OCR) and extracellular acidification rate (ECAR). Sorted mouse neutrophils were seeded at 300,000

neutrophils per well in XFe96 cell culture microplates (Agilent Technologies) precoated with poly-L-lysine. Neutrophil adherence was achieved by spinning at 200×g for 1 min followed by natural deceleration. RPMI medium was replaced with 180 μL of DMEM XF base medium, pH 7.4 (Agilent Technologies) supplemented with 10 mM glucose, 2 mM glutamine, 1 mM pyruvate (Agilent Technologies). Plates were kept for 30 min at 37°C and loaded into the Seahorse XFe96 Analyser. In the analyzer, oligomycin 2 μM, Carbonyl cyanide-4-(trifluoromethoxy) phenylhydrazone (FCCP) 1 μM, rotenone + antimycin A 0.5 μM were injected at the indicated times. Respiratory parameters were obtained as indicated: basal respiration as baseline OCR; ATP-linked respiration by subtracting the proton leak to the basal OCR; maximal OCR by calculating the difference of antimycin plus rotenone rate from FCCP rate.

Extracellular traps (ETs) assay

ETs were quantified as previously described, with some modifications (14). Briefly, freshly isolated neutrophils with 90-95% viability, were incubated with 100nM 4b-phorbol 12-myristate 13-acetate (PMA) or only media, in 96-well plates at 37 °C for 3h. Cells were then washed twice and stained with phenotyping markers in the presence of a cell impermeable DNA binding SYTOX Green dye (Thermo Fisher Scientific) at 0.5μM. Samples were washed twice and immediately analyzed by flow cytometry. Other cell types in the isolated intrahepatic leucocytes (IHLs) are similarly assayed for ETs. To account for differences in basal levels of ETosis across samples, ETosis is expressed as induction of NETosis: (% SYTOX Green+ PMA / % of control).

Intracellular ROS quantification

Intracellular ROS were detected by flow cytometry using CM-H2DCFDA (Thermo Fisher Scientific, C6827). IHLs were loaded with 5 μM CM-H2DCFDA at 37 °C for 30 min. *In vitro*-induced oxidative activity was obtained upon stimulation of cells with 50nM PMA at 37 °C for 30 min. Samples were washed twice in PBS + BSA 0.5% before FACS analysis.

References:

1. Umeshappa CS, Sole P, Surewaard BGJ, et al. Liver-specific T regulatory type-1 cells program local neutrophils to suppress hepatic autoimmunity via CRAMP. Cell Rep 2021;34:108919.
2. Flores Molina M, Fabre T, Cleret-Buhot A, et al. Visualization, Quantification, and Mapping of Immune Cell Populations in the Tumor Microenvironment. J Vis Exp 2020.
3. Flores Molina M, Abdelnabi MN, Mazouz S, et al. Distinct spatial distribution and roles of Kupffer cells and monocyte-derived macrophages in mouse acute liver injury. Front Immunol 2022;13:994480.
4. Robinson MD, Oshlack A. A scaling normalization method for differential expression analysis of RNA-seq data. Genome Biol 2010;11:R25.
5. Law CW, Chen Y, Shi W, Smyth GK. voom: Precision weights unlock linear model analysis tools for RNA-seq read counts. Genome Biol 2014;15:R29.
6. Ritchie ME, Phipson B, Wu D, et al. limma powers differential expression analyses for RNA-sequencing and microarray studies. Nucleic Acids Res 2015;43:e47.
7. Khoyratty TE, Ai Z, Ballesteros I, et al. Distinct transcription factor networks control neutrophil-driven inflammation. Nat Immunol 2021;22:1093-1106.
8. Zhou G, Soufan O, Ewald J, Hancock REW, Basu N, Xia J. NetworkAnalyst 3.0: a visual analytics platform for comprehensive gene expression profiling and meta-analysis. Nucleic Acids Res 2019;47:W234-W241.
9. Raudvere U, Kolberg L, Kuzmin I, et al. g:Profiler: a web server for functional enrichment analysis and conversions of gene lists (2019 update). Nucleic Acids Res 2019;47:W191-W198.
10. Mi H, Muruganujan A, Ebert D, Huang X, Thomas PD. PANTHER version 14: more genomes, a new PANTHER GO-slim and improvements in enrichment analysis tools. Nucleic Acids Res 2019;47:D419-D426.
11. Subramanian A, Tamayo P, Mootha VK, et al. Gene set enrichment analysis: a knowledge-based approach for interpreting genome-wide expression profiles. Proc Natl Acad Sci U S A 2005;102:15545-15550.
12. Merico D, Isserlin R, Stueker O, Emili A, Bader GD. Enrichment map: a network-based method for gene-set enrichment visualization and interpretation. PLoS One 2010;5:e13984.
13. Li H, Handsaker B, Wysoker A, et al. The Sequence Alignment/Map format and SAMtools. Bioinformatics 2009;25:2078-2079.
14. Bankevich A, Nurk S, Antipov D, et al. SPAdes: a new genome assembly algorithm and its applications to single-cell sequencing. J Comput Biol 2012;19:455-477.
15. Gouy M, Tannier E, Comte N, Parsons DP. Seaview Version 5: A Multiplatform Software for Multiple Sequence Alignment, Molecular Phylogenetic Analyses, and Tree Reconciliation. Methods Mol Biol 2021;2231:241-260.
16. Waterhouse A, Bertoni M, Bienert S, et al. SWISS-MODEL: homology modelling of protein structures and complexes. Nucleic Acids Res 2018;46:W296-W303.
17. Monteiro LB, Davanzo GG, de Aguiar CF, Moraes-Vieira PMM. Using flow cytometry for mitochondrial assays. MethodsX 2020;7:100938.

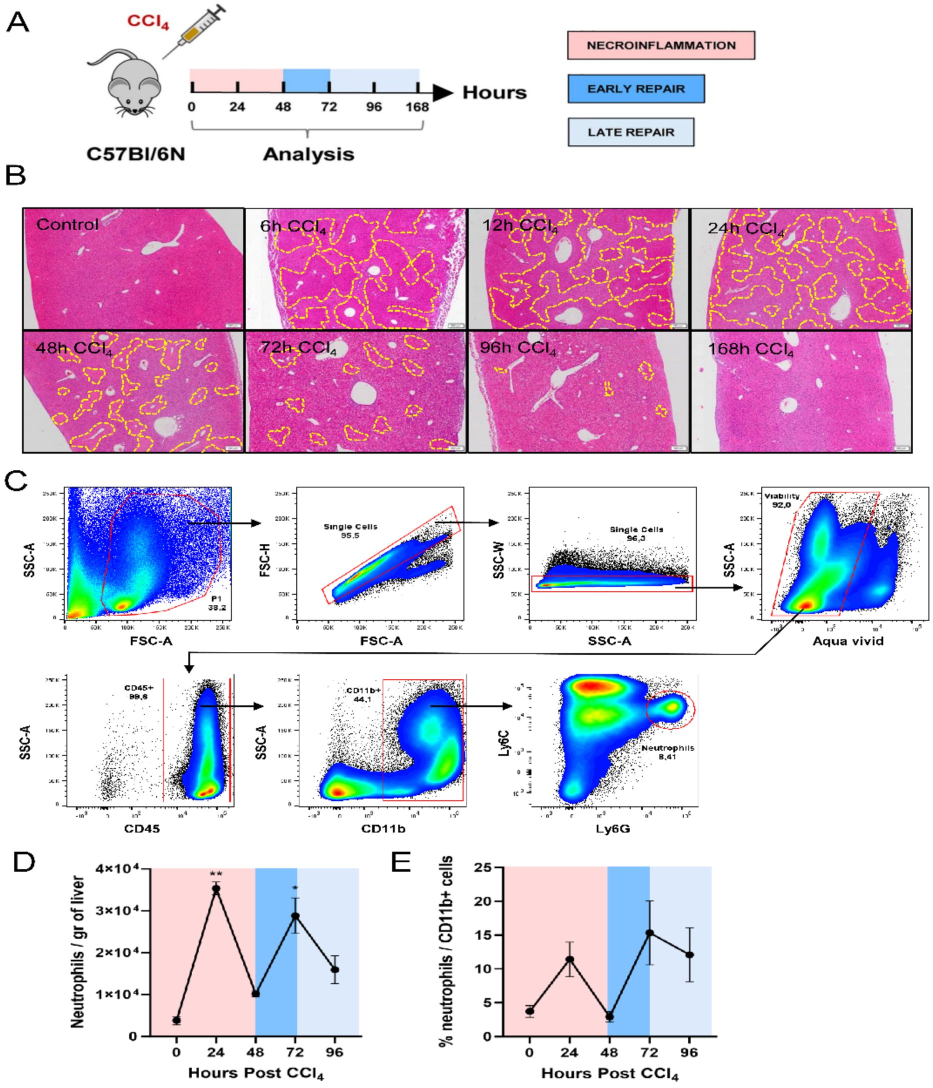

Figure S1. CCl_4-induced acute liver injury is characterized by two waves of neutrophils during necroinflammation and tissue repair. **(A)** Schematics of the experimental design delineating the phases of the wound healing response to one intraperitoneal injection of CCl_4 at 1 ml/Kg of body weight. **(B)** Representative H&E images of liver section, at 0 h-, 6 h, 12 h, 24 h, 48 h, 72 h, 96 h and 168 h following CCl_4 injection. Necrotic area was delineated manually around CV as outlined by the yellow dotted line, scale bar=200 µm. **(C)** Representative FACS plot showing the gating strategy for mouse liver neutrophils identified as $Ly6G^+$, $Ly6C^{int}$ on $CD11b^+$ cells from intrahepatic leukocytes. **(D)** Kinetics of neutrophils recruitment determined by flow cytometry represented as total counts per gram of liver or **(E)** frequencies on $CD11b^+$ population, adapted from our previous publication (3). N=4 mice per group. Data are shown as mean ± SEM. Statistical analysis was performed using one-way ANOVA on ranks followed by Dunn's Multiple Comparison Test. $^*P < 0.05$, $^{**}P < 0.01$.

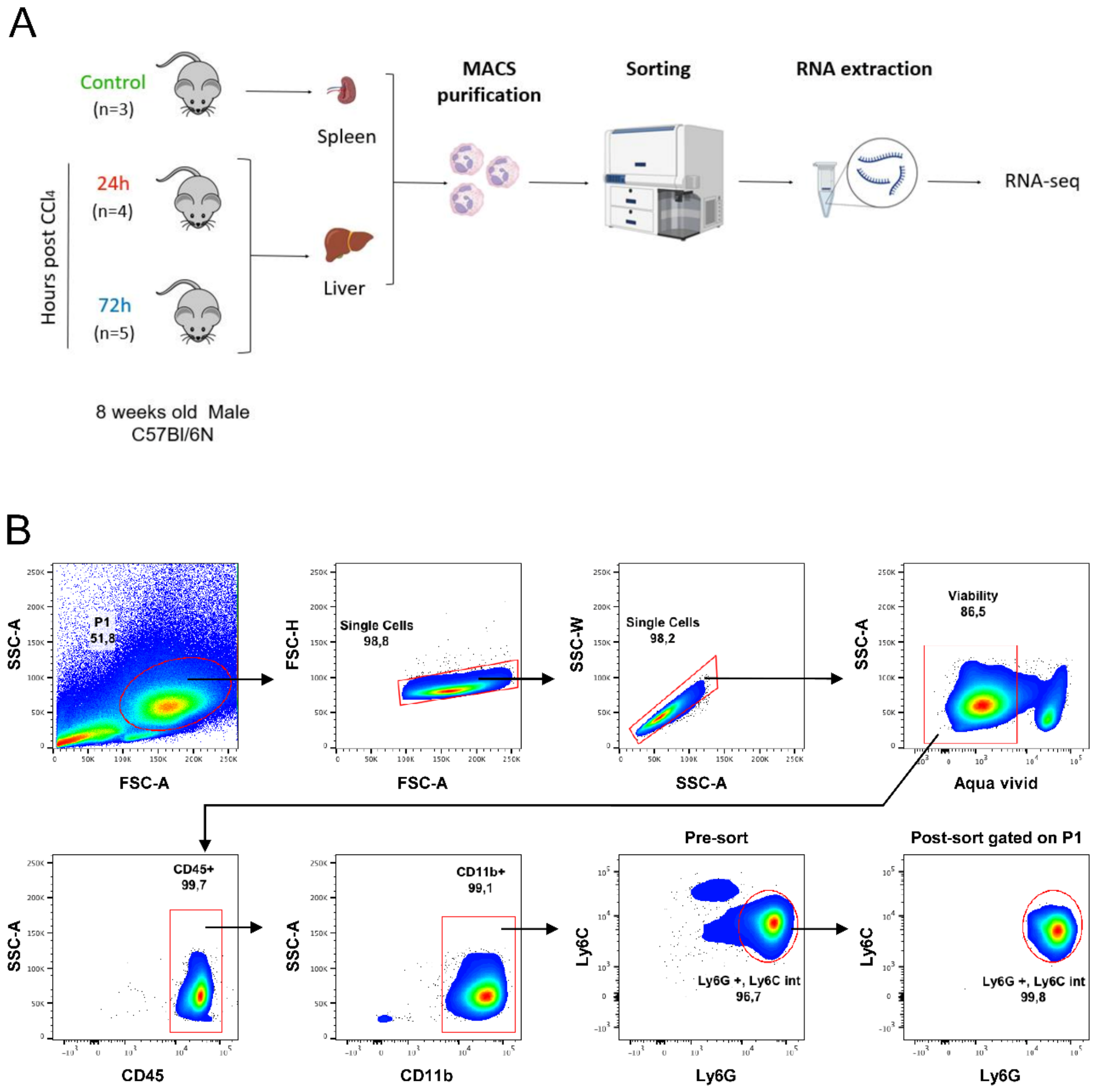

Figure S2. Neutrophils isolation method for bulk RNA-seq study. **(A)** Experimental setup scheme for bulk RNA-seq study. **(B)** Representative gating strategy for Fluorescence Activated Cell Sorting of neutrophils ($CD45^{+}CD11b^{+}Ly6G^{+}$, $Ly6C^{int}$).

A

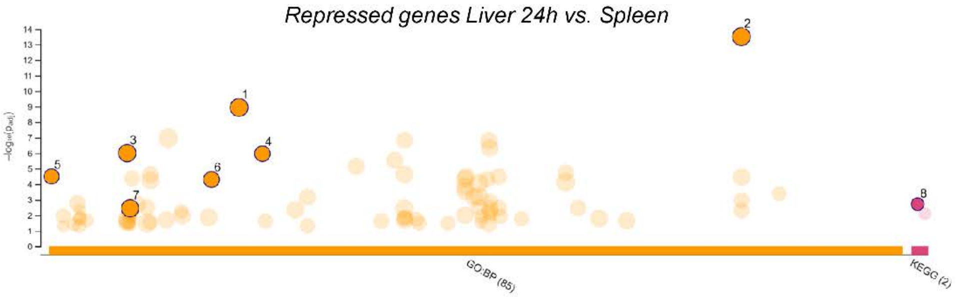

ID	Source	Term ID	Term Name	p_{adj} (query_1)
1	GO:BP	GO:0019538	protein metabolic process	1.116×10^{-9}
2	GO:BP	GO:1901564	organonitrogen compound metabolic process	3.086×10^{-14}
3	GO:BP	GO:0006810	transport	9.717×10^{-7}
4	GO:BP	GO:0022402	cell cycle process	1.070×10^{-6}
5	GO:BP	GO:0000278	mitotic cell cycle	3.161×10^{-5}
6	GO:BP	GO:0016192	vesicle-mediated transport	4.933×10^{-5}
7	GO:BP	GO:0006950	response to stress	3.508×10^{-3}
8	KEGG	KEGG:03010	Ribosome	1.872×10^{-3}

B

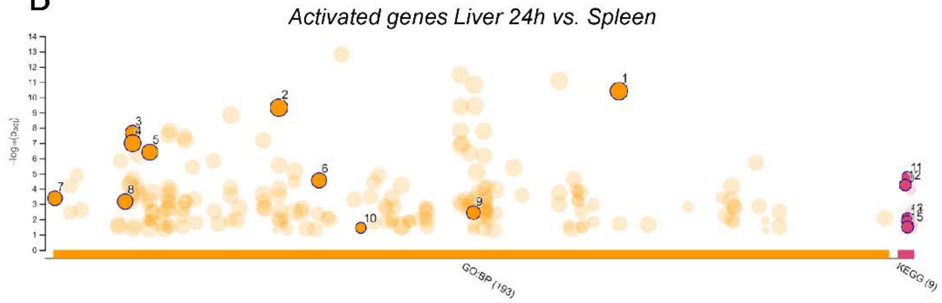

ID	Source	Term ID	Term Name	p_{adj} (query_1)
1	GO:BP	GO:0080090	regulation of primary metabolic process	3.962×10^{-11}
2	GO:BP	GO:0031323	regulation of cellular metabolic process	4.772×10^{-10}
3	GO:BP	GO:0006954	inflammatory response	2.067×10^{-8}
4	GO:BP	GO:0006950	response to stress	1.020×10^{-7}
5	GO:BP	GO:0008219	cell death	3.939×10^{-7}
6	GO:BP	GO:0034097	response to cytokine	2.793×10^{-5}
7	GO:BP	GO:0000165	MAPK cascade	4.144×10^{-4}
8	GO:BP	GO:0006629	lipid metabolic process	6.752×10^{-4}
9	GO:BP	GO:0050727	regulation of inflammatory response	3.611×10^{-3}
10	GO:BP	GO:0038066	p38MAPK cascade	3.537×10^{-2}
11	KEGG	KEGG:04668	TNF signaling pathway	1.762×10^{-5}
12	KEGG	KEGG:04064	NF-kappa B signaling pathway	5.615×10^{-5}
13	KEGG	KEGG:04657	IL-17 signaling pathway	8.596×10^{-3}
14	KEGG	KEGG:04620	Toll-like receptor signaling pathway	1.337×10^{-2}
15	KEGG	KEGG:04625	C-type lectin receptor signaling pathway	3.137×10^{-2}

Figure S3. Manhattan plot illustrating the results of over-representation analysis (ORA) on the DEG's between Liver 24h post CCl_4 neutrophils compared with spleen neutrophils. The functional terms are grouped and color-coded by data sources, i.e., biological processes (BP) in orange and KEGG in pink. Selected numbered terms in dark are detailed below the plot with their respective adjP values. **(A)** ORA on repressed **(A)** or up-regulated **(B)** DEG's from the comparison Liver 24h vs. Spleen. The functional enrichment analysis was performed using g:Profiler (version e110_eg57_p18_4b54a898) with g:SCS multiple testing correction method applying significance threshold of 0.05.

A

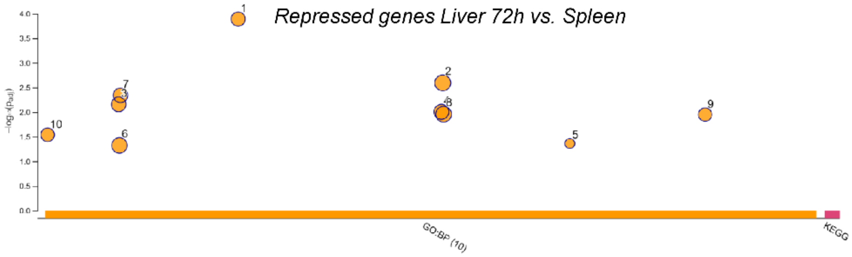

ID	Source	Term ID	Term Name	P_{adj} (query_1)
1	GO:BP	GO:0022402	cell cycle process	1.281×10^{-4}
2	GO:BP	GO:0051179	localization	2.548×10^{-3}
3	GO:BP	GO:0006952	defense response	6.949×10^{-3}
4	GO:BP	GO:0051128	regulation of cellular component organization	9.849×10^{-3}
5	GO:BP	GO:0086009	membrane repolarization	4.339×10^{-2}
6	GO:BP	GO:0006996	organelle organization	4.750×10^{-2}
7	GO:BP	GO:0007049	cell cycle	4.561×10^{-3}
8	GO:BP	GO:0051234	establishment of localization	1.100×10^{-2}
9	GO:BP	GO:1903047	mitotic cell cycle process	1.119×10^{-2}
10	GO:BP	GO:0000278	mitotic cell cycle	2.891×10^{-2}

B

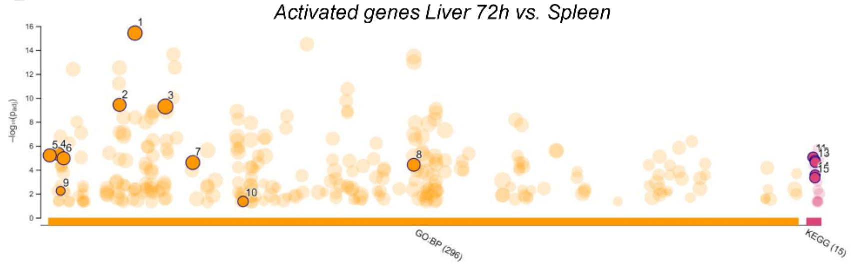

ID	Source	Term ID	Term Name	P_{adj} (query_1)
1	GO:BP	GO:0008219	cell death	3.790×10^{-16}
2	GO:BP	GO:0006954	inflammatory response	3.746×10^{-10}
3	GO:BP	GO:0010604	positive regulation of macromolecule metabolic pr...	5.139×10^{-10}
4	GO:BP	GO:0001568	blood vessel development	4.698×10^{-6}
5	GO:BP	GO:0000165	MAPK cascade	6.225×10^{-6}
6	GO:BP	GO:0001944	vasculature development	1.097×10^{-5}
7	GO:BP	GO:0016477	cell migration	2.466×10^{-5}
8	GO:BP	GO:0048514	blood vessel morphogenesis	3.805×10^{-5}
9	GO:BP	GO:0001780	neutrophil homeostasis	5.728×10^{-3}
10	GO:BP	GO:0030593	neutrophil chemotaxis	4.420×10^{-2}
11	KEGG	KEGG:04064	NF-kappa B signaling pathway	8.984×10^{-6}
12	KEGG	KEGG:04625	C-type lectin receptor signaling pathway	1.753×10^{-5}
13	KEGG	KEGG:04668	TNF signaling pathway	2.301×10^{-5}
14	KEGG	KEGG:04657	IL-17 signaling pathway	2.704×10^{-4}
15	KEGG	KEGG:04620	Toll-like receptor signaling pathway	4.560×10^{-4}

Figure S4. Manhattan plot illustrating the results of over-representation analysis (ORA) on the DEG's between Liver 72h post CCl_4 neutrophils compared with spleen neutrophils. The functional terms are grouped and color-coded by data sources, i.e., biological processes (BP) in orange and KEGG in pink. Selected numbered terms in dark are detailed below the plot with their respective adjP values. ORA on repressed **(A)** or up-regulated **(B)** DEG's from the comparison Liver 72h vs. Spleen. The functional enrichment analysis was performed using g:Profiler (version e110_eg57_p18_4b54a898) with g:SCS multiple testing correction method applying significance threshold of 0.05.

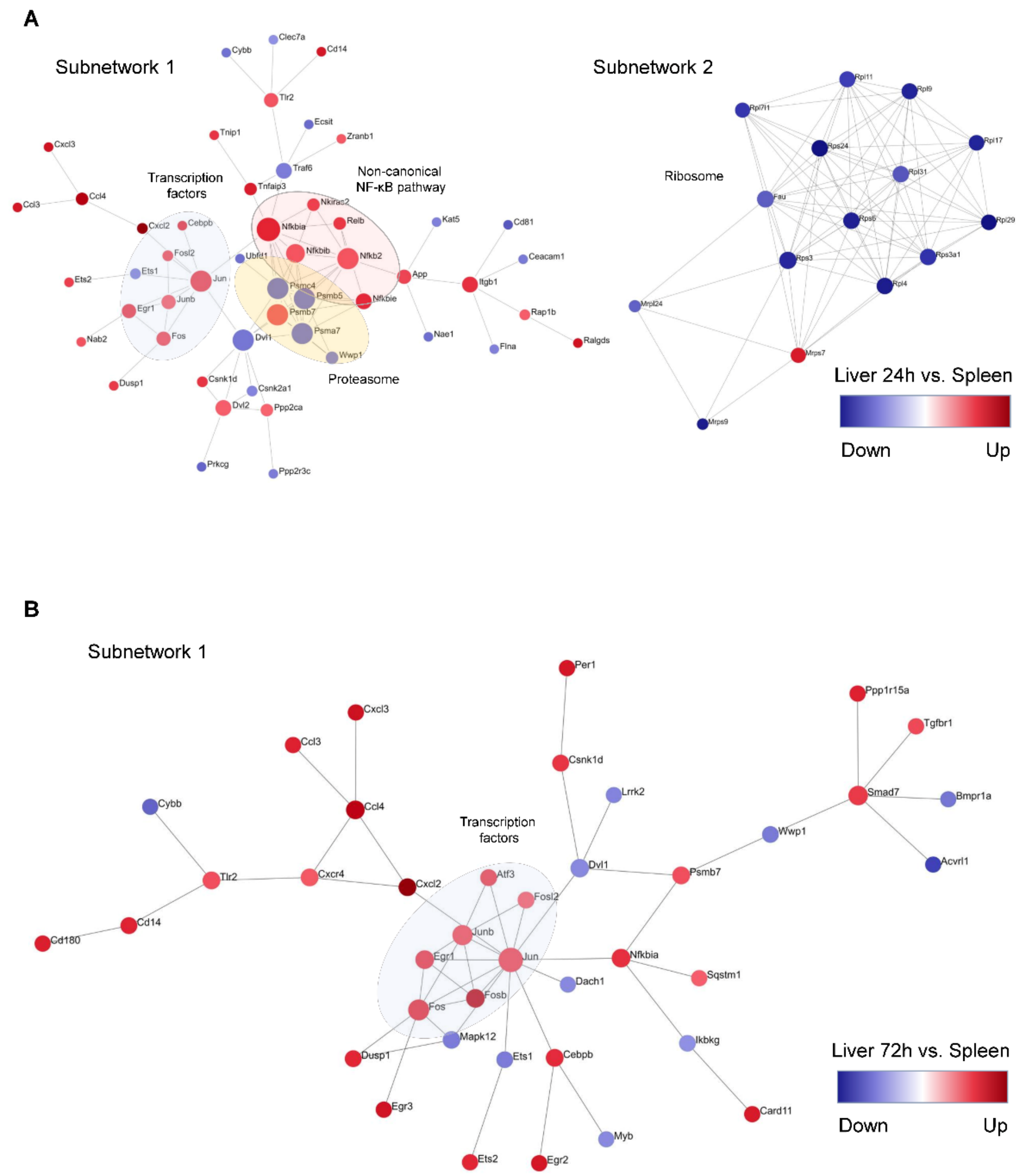

Figure S5. Protein-protein interaction (PPI) network analysis of the DEG's. **(A)** PPI analysis identified 2 networks of highly interconnected nodes from Liver 24h vs spleen neutrophils comparison, respectively correlated to the "inflammatory response" and to the "protein translation". **(B)** PPI analysis identified 1 network of highly interconnected nodes from Liver 72h vs spleen neutrophils comparison, correlated to the "inflammatory response". Colors represent the expressions of nodes, specifically "red" and "blue" indicate the nodes that are up- and down-regulated, respectively. Grades of the colors represent the expression levels. Areas of the nodes indic#ate the degrees that the nodes connect to others. PPI analysis was done through the NetworkAnalyst 3.0 platform.

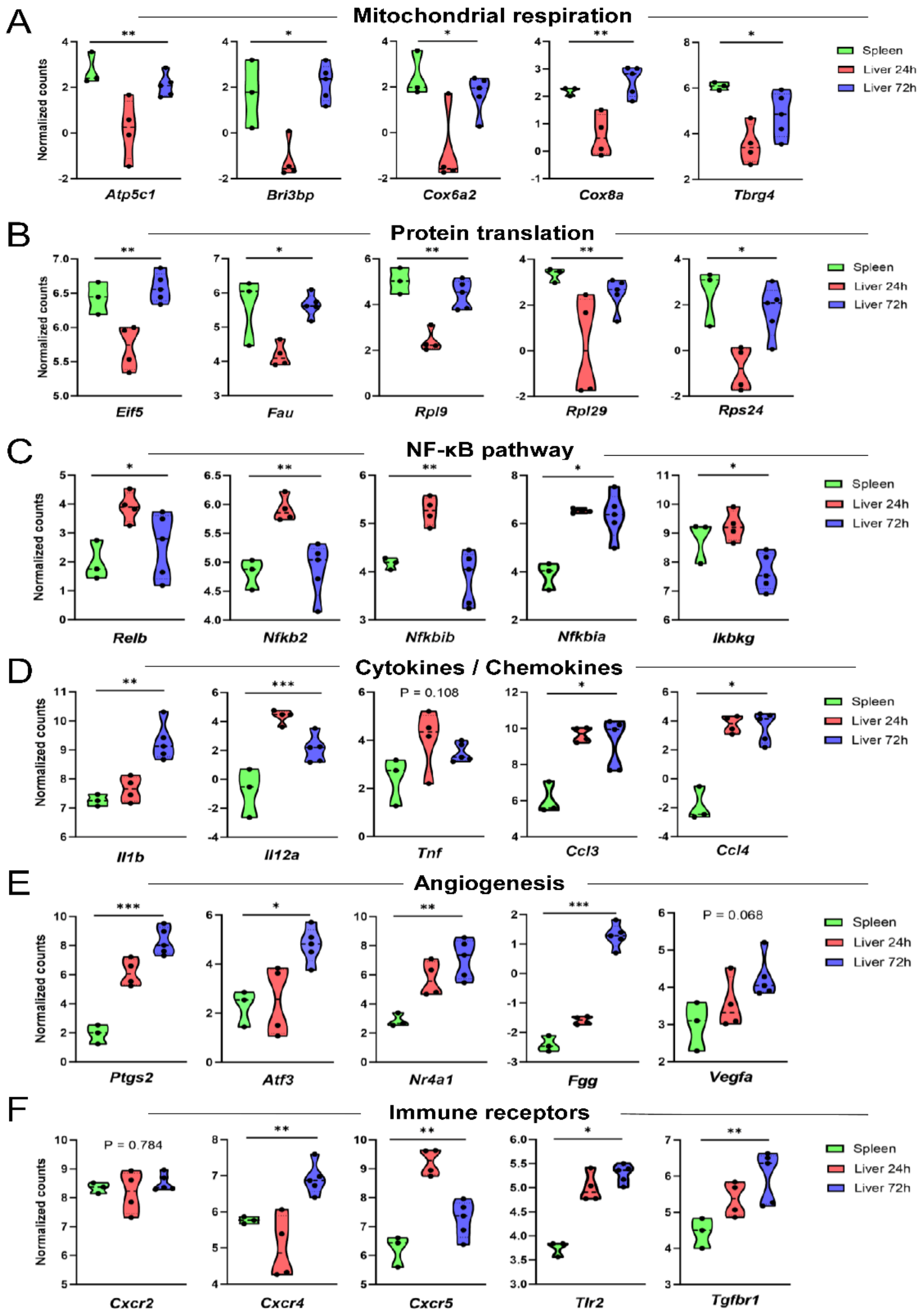

Figure S6. Violin plots showing neutrophils groups for gene expression as TMM normalized counts. Six functional gene categories are included: **(A)** Mitochondrial respiration-, **(B)** Protein translation-, **(C)** NF-κB pathway-, **(D)** Cytokines-, **(E)** Angiogenesis- and **(F)** Immune receptors. Each dot represents a sample. Statistical analysis was performed using One Way ANOVA on ranks. *$P < 0.05$, **$P < 0.01$, ***$P < 0.001$.

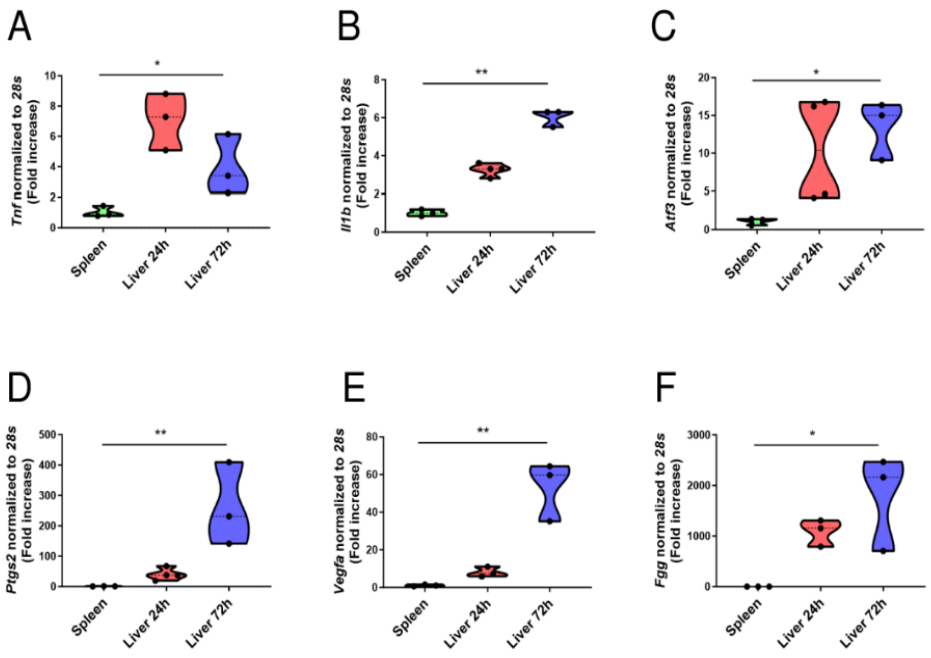

Figure S7. RT-qPCR validation**. (A-F)** Relative gene expressions for six selected genes were determined by qPCR on FACS sorted neutrophils RNA. The mRNA expression data represent fold increase relative to spleen controls and was normalized to 28s. N=3-4 mice per group. Data are shown as mean ± SEM. Statistical analysis was performed using One Way ANOVA on ranks. **P* < *0.05*, ***P* < *0.01*.

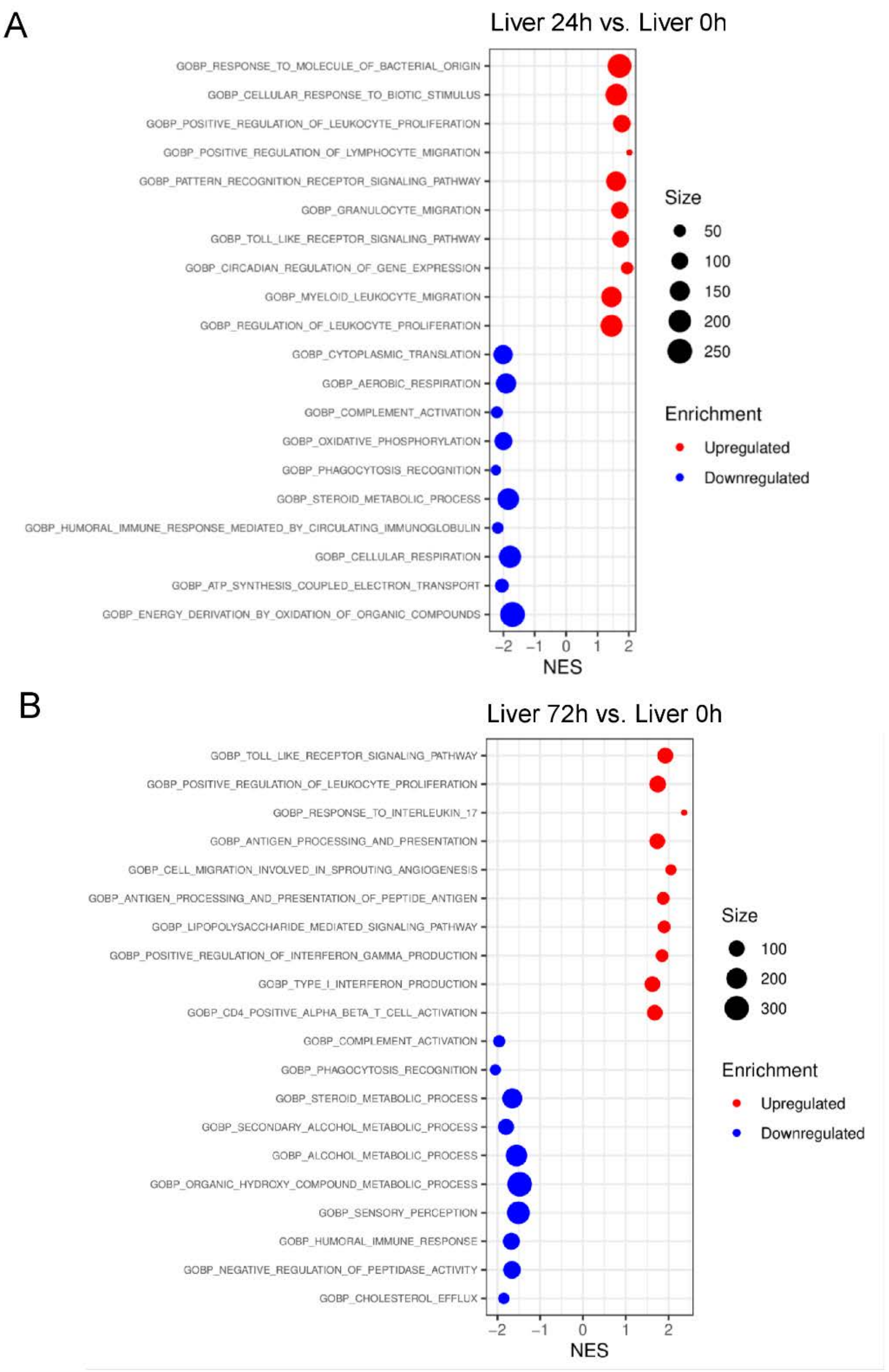

Figure S8. GSEA demonstrates functionally distinct 24h- and 72h post-injury liver compared to naïve liver neutrophils extracted from publicly available datasets. GSEA dot plots of Liver 24h vs. Liver0h and **(A)** Liver 72h vs. Liver0h **(B)** comparison. The figures show the significant top 10 positively and the top 10 negatively enriched GO terms. Size refers to the number of genes associated with each GO biological process. Terms are ranked in the figure by the adjusted p-value. Node color represents the normalized enrichment scores (NES).

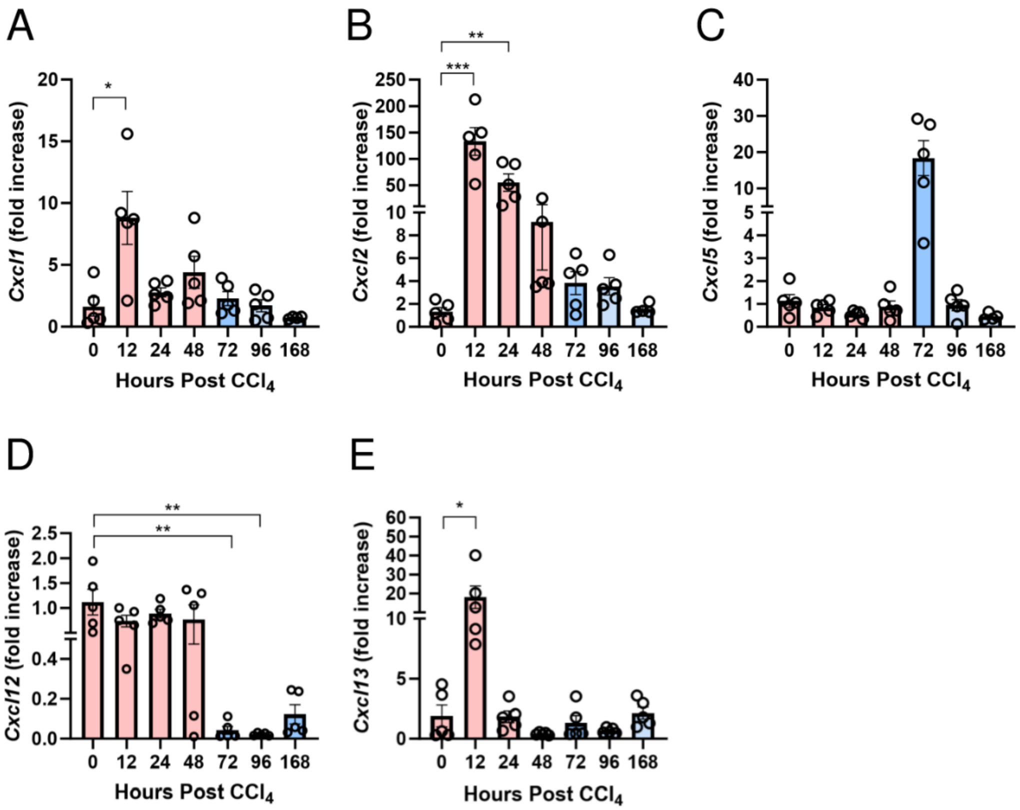

Figure S9. Relative gene expression levels of *Cxcl1* **(A)**, *Cxcl2* **(B)**, *Cxcl5* **(C)**, *Cxcl13* **(D)** *and Cxcl12* **(E)** respectively, as determined by qPCR on bulk liver tissue. The mRNA expression data represent fold increase relative to 0 h controls and was normalized to 28s. (N=5 mice per group). Data are shown as mean ± SEM. Statistical analysis was performed using One Way ANOVA on ranks and Dunn's Multiple Comparison Test. *$P < 0.05$, **$P < 0.01$, ***$P < 0.001$, (Related to Fig. 6)

Supplemental Tables

Table S1. List of flow cytometry antibodies used in the study.

Antibody	Clone	Supplier	Catalog number
CD3-PE-CF594	145-2C11	BD	562286
CD11c-BV711	N418	BioLegend	117349
CD11b-BV421	M1/70	BD	562605
CD45-BV650	30-F11	BD	563410
Ly6C-PECF594	AL-21	BD	562728
Ly6C-APC	AL-21	BD	560595
Ly6G-A488	1A8	BioLegend	127625
Ly6G-APC-e780	1A8	eBioscience	47-9668-82
CD184-APC-R700	2B11/CXCR4	BD	565522
CD185-PE	2G8/CXCR5	BD	551959
Blr1-PE	C-3/CXCR5	SCB	sc-373775 PE
Puromycin-AF647	12D10	Millipore-Sigma	MABE343-AF647
CD170 (Siglec-F)-BUV737	1RNM44N	eBioscience	367-1702-82

Table S2. List of immunofluorescence antibodies.

Immunofluorescence Primary Abs	Company	Clone	Catalog	State, country
MPO	R&D Systems	Polyclonal Goat	AF3667	Canada
IBA1	Fujifilm WAKO Chemicals	Rabbit Polyclonal	019-19741	USA
αSMA	Sigma Aldrich	1A4	A2547-100UL	Canada
Desmin	Invitrogen	Rabbit Polyclonal	PA5-16705	Canada
**Immunofluorescence Secondary Abs**				
Donkey anti-goat A568	Invitrogen	Donkey Polyclonal	A-11057	Canada
Donkey anti-rabbit A647	Invitrogen	Donkey Polyclonal	A-31573	Canada
Donkey anti-mouse, DyLight™ 755	Invitrogen	Donkey Polyclonal	SA5-10171	Canada

Table S3. List of primer sequences used in the study.

Gene	Forward, 5'-3'	Reverse, 5'-3'
28s	CGAGATTCCCACTGTCCCTA	GGGGCCTCCCACTTATTCTA
Atf3	GAGGATTTTGCTAACCTGACACC	TTGACGGTAACTGACTCCAGC
Fgg	GACGGCATTATTTGGGCGAC	AACGTCTCCAGCCTGTTTGG
Cxcl1	GGATTCACCTCAAGAACATCCAG	ATCTTTTGGACAATTTTCTGAACC
Cxcl2	GAGCTTGAGTCTGACGCCCCCAGG	GTTAGCCTTGCCTTTGTTCAGTATC
Cxcl5	GCATTTCTGTTGCTGTTCACGCTG	CCTCCTTCTGGTTTTTCAGTTTAGC
Cxcl12	GTCTAAGCAGCGATGGGTTC	GAATAAGAAAGCACACGCTGC
Cxcl13	GGCCACGGTATTCTGGAAGC	ACCGACAACAGTTGAAATCACTC
Ptgs2	CCA CTT CAA GGG AGT CTG GA	AGT CAT CTG CTA CGG GAG GA
Tnf	ACT CCA GGC GGT GCC TAT GT	GTG AGG GTC TGG GCC ATA GAA
Vegfa	CAG GCT GCT GTA ACG ATG AA	TTT GAC CCT TTC CCT TTC CT

Table S4. DEGs, liver neutrophils from 24h post-injury compared to control (splenic neutrophils).

Table S5. DEGs, liver neutrophils from 72h post-injury compared to control (splenic neutrophils).

Table S6. Comprehensive list of all statistically significant GSEA genesets enriched in liver neutrophils from 24h post-injury compared to control (splenic neutrophils). A total of 147 subsets from the Gene Ontology: Biological Processing geneset were enriched in livers neutrophils from 24h post-injury compared to control. A false discovery rate (FDR) < 0.2 and an adjusted p-value < 0.05 were used.

Table S7. Comprehensive list of all statistically significant GSEA genesets enriched in liver neutrophils from 72h post-injury compared to control (splenic neutrophils). A total of 205 subsets from the Gene Ontology: Biological Processing geneset were enriched in livers neutrophils from 72h post-injury compared to control. A false discovery rate (FDR) < 0.2 and an adjusted p-value < 0.05 were used.

Table S8. DEGs, liver neutrophils from 24h post-injury compared to naïve liver neutrophils extracted from the GSE180824 datasets.

Table S9. DEGs, liver neutrophils from 72h post-injury compared to naïve liver neutrophils extracted from the GSE180824 datasets.

Table S10. Comprehensive list of all statistically significant GSEA genesets enriched in liver neutrophils from 24h post-injury compared to naïve liver neutrophils extracted from the GSE180824 datasets. A total of 127 subsets from the Gene Ontology: Biological Processing geneset were enriched in liver neutrophils from 24h post-injury compared to control. A false discovery rate (FDR) < 0.1 and an adjusted p-value < 0.05 were used.

Table S11. Comprehensive list of all statistically significant GSEA genesets enriched in liver neutrophils from 72h post-injury compared to naïve liver neutrophils extracted from the GSE180824 datasets. A total of 85 subsets from the Gene Ontology: Biological Processing geneset were enriched in liver neutrophils from 72h post-injury compared to control. A false discovery rate (FDR) < 0.1 and an adjusted p-value < 0.05 were used.
